# Anthocyanins, Anthocyanin-Rich Berries, and Cardiovascular Risks: Systematic Review and Meta-Analysis of 44 Randomized Controlled Trials and 15 Prospective Cohort Studies

**DOI:** 10.3389/fnut.2021.747884

**Published:** 2021-12-15

**Authors:** Lin Xu, Zezhong Tian, Hong Chen, Yimin Zhao, Yan Yang

**Affiliations:** ^1^School of Public Health (Shenzhen), Sun Yat-sen University, Guangzhou, China; ^2^Guangdong Provincial Key Laboratory of Food, Nutrition, and Health, Sun Yat-sen University, Guangzhou, China; ^3^Guangdong Engineering Technology Center of Nutrition Transformation, Sun Yat-sen University, Guangzhou, China; ^4^China-DRIs Expert Committee on Other Food Substances, Sun Yat-sen University, Guangzhou, China

**Keywords:** anthocyanin, berry, cardiovascular diseases, meta-analysis, randomized controlled trial, prospective cohort study

## Abstract

**Objective:** The associations between intake of anthocyanins and anthocyanin-rich berries and cardiovascular risks remained to be established. We aimed to quantitatively summarize the effects of purified anthocyanins and anthocyanin-rich berries on major surrogate markers of cardiovascular diseases (CVDs) and the longitudinal associations between dietary anthocyanins and CVD events.

**Methods:** Meta-analysis of randomized controlled trials (RCTs) and prospective cohort studies.

**Results:** We included 44 eligible RCTs and 15 prospective cohort studies in this study. Pooled analysis of RCTs showed that purified anthocyanin supplementation could significantly reduce blood LDL cholesterol (weighted mean difference (WMD): −5.43 mg/dL, 95% CI: −8.96, −1.90 mg/dL; *p* = 0.003) and triglyceride (WMD: −6.18 mg/dL, 95% CI: −11.67, −0.69 mg/dL; *p* = 0.027) while increase HDL cholesterol (WMD: 11.49 mg/dL, 95% CI: 7.43, 15.55 mg/dL; *p* < 0.001) concentrations. Purified anthocyanins also markedly decreased circulating tumor necrosis factor alpha (WMD: −1.62 pg/mL, 95% CI: −2.76, −0.48 pg/mL; *p* = 0.005) and C-reactive protein (WMD: −0.028 mg/dL, 95% CI: −0.050, −0.005 mg/dL; *p* = 0.014). Besides, administration of anthocyanin-rich berries could significantly lower blood total cholesterol (WMD: −4.48 mg/dL, 95% CI: −8.94, −0.02 mg/dL; *p* = 0.049) and C-reactive protein (WMD: −0.046 mg/dL, 95% CI: −0.070, −0.022 mg/dL; *p* < 0.001). Neither purified anthocyanins nor anthocyanin-rich berries could cause any substantial improvements in BMI, blood pressure, or flow-mediated dilation. In addition, meta-analysis of prospective cohort studies suggested that high dietary anthocyanins were related to lower risk of coronary heart disease (CHD) (relative risk (*RR*): 0.83, 95% CI: 0.72, 0.95; *p* = 0.009), total CVD incidence (*RR*: 0.73, 95% CI: 0.55, 0.97; *p* = 0.030), and total CVD deaths (*RR*: 0.91, 95% CI: 0.87, 0.96; *p* < 0.001).

**Conclusion:** Habitual intake of anthocyanins and anthocyanin-rich berries could protect against CVDs possibly *via* improving blood lipid profiles and decreasing circulating proinflammatory cytokines.

**Systematic Review Registration:** https://www.crd.york.ac.uk/PROSPERO, identifier: CRD42020208782.

## Introduction

Cardiovascular diseases (CVDs) remain the leading cause of premature death globally, which have exerted persistent and tremendous burdens on healthcare systems in the recent decades (1). Predominant risk factors of CVDs include but not restrict to overweight, hypertension, and elevated blood atherogenic lipoproteins (2). It is estimated that successful management of blood lipids could lead to about 30% less CVD events among Chinese hypertensive adults (3). Besides, CVDs could result in over 60% deaths in patients with diabetes, and they suffered from a worse prognosis for survival than patients with CVD without diabetes (4). Circulating biomarkers of chronic low-grade inflammation, such as C-reactive protein (CRP) and tumor necrosis factor alpha (TNF-α), could also serve as independent predictors of future CVD events (5).

Diet modification is the pivotal strategy for CVD prevention (6, 7). Firm epidemiological evidence has established strong inverse associations between CVD risks and dietary intake of plant foods and plant-based bioactive constitutes (8–10). Anthocyanins are polyphenolic pigments, which are rich in dark-colored plant foods including berries, grapes, onions, and black rice (11, 12). Increasing research interest has focused on the health benefits of anthocyanins and anthocyanin-rich foods (13–15). Owing to rich hydroxyl groups in their chemical structures, anthocyanins also represent one of the largest families of phenolic pigments with antioxidant and anti-inflammatory properties (16). Habitual consumption of anthocyanins and anthocyanin-rich foods was suggested to reduce the risks of various chronic diseases including CVD, neuroinflammatory process, and liver steatosis (17). A previous meta-analysis of prospective studies found that frequent intake of anthocyanin-rich foods was related to 9% lower risk of coronary heart disease (CHD) (18). Both clinical and preclinical investigations have demonstrated strong lipid-lowering effects of anthocyanins (19, 20). In addition, anthocyanin intake could substantially improve endothelial function and alleviate arterial stiffness among subjects with high cardiovascular risks (21). However, the effects of anthocyanins on adiposity, blood pressure, and chronic low-grade inflammation were still conflicting (20, 22, 23). Our recent study unraveled that anthocyanins could dose-dependently reduce blood ceramides, newly identified predictors of CVDs, in the dyslipidemia subjects (24).

Berries comprised of about 10% of total fruit consumption in the United States (25) and served as the main dietary sources of anthocyanins regardless of commercialized anthocyanin supplements (11). Although the anthocyanin contents vary dramatically across berry species and are profoundly influenced by cultivation, preservation, and processing (11), blueberry, cranberry, bilberry, and blackcurrant basically rank the most plentiful in anthocyanins among berry fruits, which could contain 100 to 200 mg anthocyanins per 100 g edible portions (Supplementary Table 1). In turn, anthocyanins composed the largest proportions of bioactive polyphenols in ripe berries and were suggested to make the greatest impacts on the physiological improvements from berry intake (11, 26). Regular consumption of anthocyanins and anthocyanin-rich berries has been widely recommended due to their potential cardioprotective benefits (26, 27), even though the associations and causal effects were still elusive (28, 29). Therefore, we aimed to quantitatively summarize current eligible randomized controlled trials (RCTs) and prospective cohort studies to investigate the associations of anthocyanins and major anthocyanin-rich berries with cardiovascular health in this study.

## Materials and Methods

The present meta-analysis was reported according to the Preferred Reporting Items for Systematic Reviews and Meta-Analyses (PRISMA) Statement (30). This study has been registered at the International Prospective Register of Systematic Reviews (PROSPERO, registration ID: CRD42020208782).

### Literature Search

Two investigators (LX and HC) searched the PubMed, Embase, and Cochrane Library for eligible studies up to December 31, 2020. Literature search for RCTs and prospective cohort studies were conducted independently. Because we did not identify any RCTs reporting CVD events in the preliminary search, we alternatively focused on the major surrogate markers of CVD in the meta-analysis of RCTs. For RCTs, the search terms were anthocyanins or anthocyanin-rich berries combined with major CVD risk factors including adiposity, blood pressure, blood lipids, and inflammation (see Online Supplementary Materials for details). For prospective cohort studies, the search terms were anthocyanins combined with fatal or non-fatal CVD events including CHD, stroke, total CVD incidence, and total CVD mortality (see Online Supplementary Materials for details). Because the anthocyanin intakes in observational studies were generally derived from various food items in diet records or food frequency questionnaire (FFQ) in our preliminary literature search, we only analyzed the relationship between anthocyanin intake and CVD events regardless of their dietary sources. We also searched reviews and meta-analysis articles concerning the effects of anthocyanins and anthocyanin-rich berries on cardiovascular health. Literature search was restricted to those published in English. We screened the titles and abstracts of all retrieved publications and then determined the eligibility *via* checking the full text.

### Study Inclusion and Exclusion

Two investigators (LX and HC) independently performed study inclusion and exclusion. Any discrepancies were resolved by discussion with other research team members until a consensus was reached. For RCTs, studies were included if they meet the following criteria: (1) were either parallel- or crossover-designed; (2) conducted in adults; (3) with a intervention duration longer than 2 weeks; (4) used purified anthocyanins or anthocyanin-rich berries including blueberry, cranberry, bilberry, and blackcurrant as the intervention approach; (5) adopted placebo or other adequate controls as the comparators; and (6) provided sufficient data for calculating changes in any of the following CVD biomarkers before and after intervention: BMI, systolic blood pressure (SBP), diastolic blood pressure (DBP), flow-mediated dilation (FMD), total cholesterol (TC), LDL cholesterol (LDL-C), HDL cholesterol (HDL-C), triglyceride (TG), CRP, and TNF-α. Studies were excluded if they (1) were acute feeding trials; (2) conducted in pregnant or lactating women, or critically ill patients (e.g., subjects with advanced cancer, end-stage cardiac insufficiency, or end-stage nephropathy); (3) had a multifactorial design; and (4) used crude plant or herb extractives as the intervention approach making it difficult to isolate the effects of anthocyanins or anthocyanin-rich berries.

For prospective cohort studies, studies were included if they (1) were prospective cohort studies; (2) conducted in adults; (3) reported baseline dietary anthocyanin intake as the exposure; (4) reported fatal or non-fatal CVD events as the outcome, including CHD incidence and mortality, stroke incidence and mortality, total CVD incidence, and total CVD mortality; and (5) provided relative risk (RR) or hazard ratio (HR) with corresponding 95% confidence intervals (CIs) or sufficient data to calculate them. Studies were excluded if they were case-control or retrospective studies.

### Quality Assessment

Quality assessments of eligible RCTs and prospective cohort studies were performed according to the National Heart, Lung, and Blood Institute (NHLBI) Quality Assessment of Controlled Intervention Studies and the NHLBI Quality Assessment Tool for Observational Cohort and Cross-Sectional Studies, respectively. A study was considered as high quality if it met at least 11 of the 14 criteria (about 80%), otherwise it was regarded as low to moderate quality.

### Statistical Analysis

We estimated heterogeneity among studies using the Cochrane's Q test, and a *p-*value < 0.1 or a *I*^2^ statistic >50% indicated substantial between-study heterogeneity. Pooled estimates were calculated using the DerSimonian–Laird random-effects model to address potential between-study heterogeneity. Statistical significance set at a *p-*value < 0.05. For RCTs, crossover studies were treated as parallel studies in a way that each intervention phase was treated as an independent arm of a parallel study. For prospective cohort studies, HRs were treated as RRs. To explore the sources of potential between-study heterogeneity, we performed pre-specified subgroup analysis stratified by study characteristics. We evaluated the robustness of pooled estimates *via* leave-one-out sensitivity analysis. We assessed the publication bias using funnel plots and also the Begg's tests. The trim and fill methods were used to correct theoretically missing studies, if any. All statistical analyses were performed in Stata/SE version 16.0 (College Station, Texas, US).

## Results

### Study Characteristics

#### CTs

We identified a total of 44 eligible RCTs consisting of 52 comparison groups and 2,353 subjects in the present meta-analysis (Supplementary Figure 1). Detailed characteristics of included studies can be found in Supplementary Table 2. Briefly, 15 of the included studies investigated the effects of purified anthocyanins, all of which were produced from berries. For the remaining anthocyanin-rich berry studies, interventions were blueberry in 13 studies, cranberry in 12 studies, bilberry in three studies, and blackcurrant in one study. Seven of the 44 studies were crossover trials with the rest parallel-designed. Most studies were conducted in Asia (*n* = 12), Europa (*n* = 14), and the United States (*n* = 15). The intervention durations ranged from 2 weeks to 24 months with a median of 8 weeks. Thirty-one of the included studies recruited subjects that were at high risks of CVDs such as patients with obesity, dyslipidemia, diabetes, and history of CVDs. Nearly half of the included studies (*n* = 21) clearly claimed that they received research grants from berry industry or industry associations.

#### Prospective Cohort Studies

We included 15 eligible prospective cohort studies including 16 independent cohorts and 5,54,638 subjects in the present meta-analysis (Supplementary Figure 2). Briefly, seven of the included cohort studies were conducted in the United States with another three in Australia and four in Europa. The follow-up periods ranged from 4.3 to 24 years with a median of 12 years. Most of the included cohort studies used FFQ to assess dietary anthocyanin intake and only three of them used dietary records (31–33) (see Supplementary Table 3 for detailed study characteristics).

### Study Quality

#### RCTs

Allocation concealment was adequate in 35 of the 44 included RCTs (Supplementary Tables 4, 5). Group assignment was sufficiently blind to both participants and clinical investigators in 33 studies. The overall dropout rates at end point were <20% in 41 studies. However, only 19 studies used adequate methods of randomization whereas 34 studies did not blind researchers assessing the outcomes to group assignment. In summary, 24 of the 44 included studies were rated as high quality with the others as low to moderate quality.

#### Prospective Cohort Studies

All the included cohort studies prospectively measured dietary anthocyanins intake prior to the ascertainment of CVD events, clearly defined the dietary assessment methods, and statistically adjusted for key potential confounding covariates (e.g., age and gender) (Supplementary Tables 6, 7). However, most included cohort studies did not report sample size justification, power estimation (12 of 15), or whether the outcome assessor was blinded to the exposure status of subjects (10 of 15). In summary, 12 of the 15 included prospective cohort studies were rated as high quality.

### Pooled Effects of Anthocyanins and Anthocyanin-Rich Berries on BMI

We did not find any significant effects of purified anthocyanins (WMD: 0.07 kg/m^2^, 95% CI: −0.09, 0.23 kg/m^2^; *p*_difference_ = 0.357; *I*^2^ = 0.0%; 15 comparisons; 901 subjects); Supplementary Table 8 or anthocyanin-rich berries (WMD: 0.06 kg/m^2^; 95% CI: −0.03, 0.15 kg/m^2^; *p*_difference_ = 0.202; *I*^2^ = 0.0%; 13 comparisons; 498 subjects) on BMI. Interestingly, subgroup analysis showed that cranberry administration could slightly but significantly reduce BMI (WMD: −0.30 kg/m^2^, 95% CI: −0.57, −0.02 kg/m^2^; *p*_difference_ = 0.035; *I*^2^ = 0.0%; three comparisons; 111 subjects).

### Pooled Effects of Anthocyanins and Anthocyanin-Rich Berries on Blood Pressure and FMD

Seventeen comparison groups including 995 subjects evaluated the effects of purified anthocyanins on blood pressure (Supplementary Tables 9, 10). Supplementation of purified anthocyanins did not cause any significant effects on SBP (WMD: −0.11 mmHg; 95% CI: −1.65, 1.44 mmHg; *p*_difference_ = 0.832; *I*^2^ = 0.0%) or DBP (WMD: 0.74 mmHg; 95% CI: −0.25, 1.72 mmHg; *p*_difference_ = 0.143; *I*^2^ = 0.0%). We observed similar results in the effects of anthocyanin-rich berries on SBP (WMD: −0.64 mmHg; 95% CI: −1.82, 0.53 mmHg; *p*_difference_ = 0.284; *I*^2^ = 77.2%; 20 comparisons; 883 subjects) and DBP (WMD: −0.96 mmHg; 95% CI: −2.13, 0.21 mmHg; *p*_difference_ = 0.107; *I*^2^ = 79.5%; 20 comparisons; 885 subjects).

Because we identified only one eligible study that reported the effects of purified anthocyanins on FMD (34), we did not perform subsequent pooled analysis and subgroup analysis. Anthocyanin-rich berry intake had no improvement in FMD (WMD: 1.20 %, 95% CI: −0.21, 2.60 %; *p*_difference_ = 0.096; *I*^2^ = 98.7%; six comparisons; 263 subjects; Supplementary Table 11) except for blackcurrant (WMD: 1.78 %, 95% CI: 0.67, 2.90 %; *p*_difference_ = 0.002; *I*^2^ = 0.0%; two comparisons; 64 subjects).

### Pooled Effects of Anthocyanins and Anthocyanin-Rich Berries on Blood Lipids

**Figure 1:**
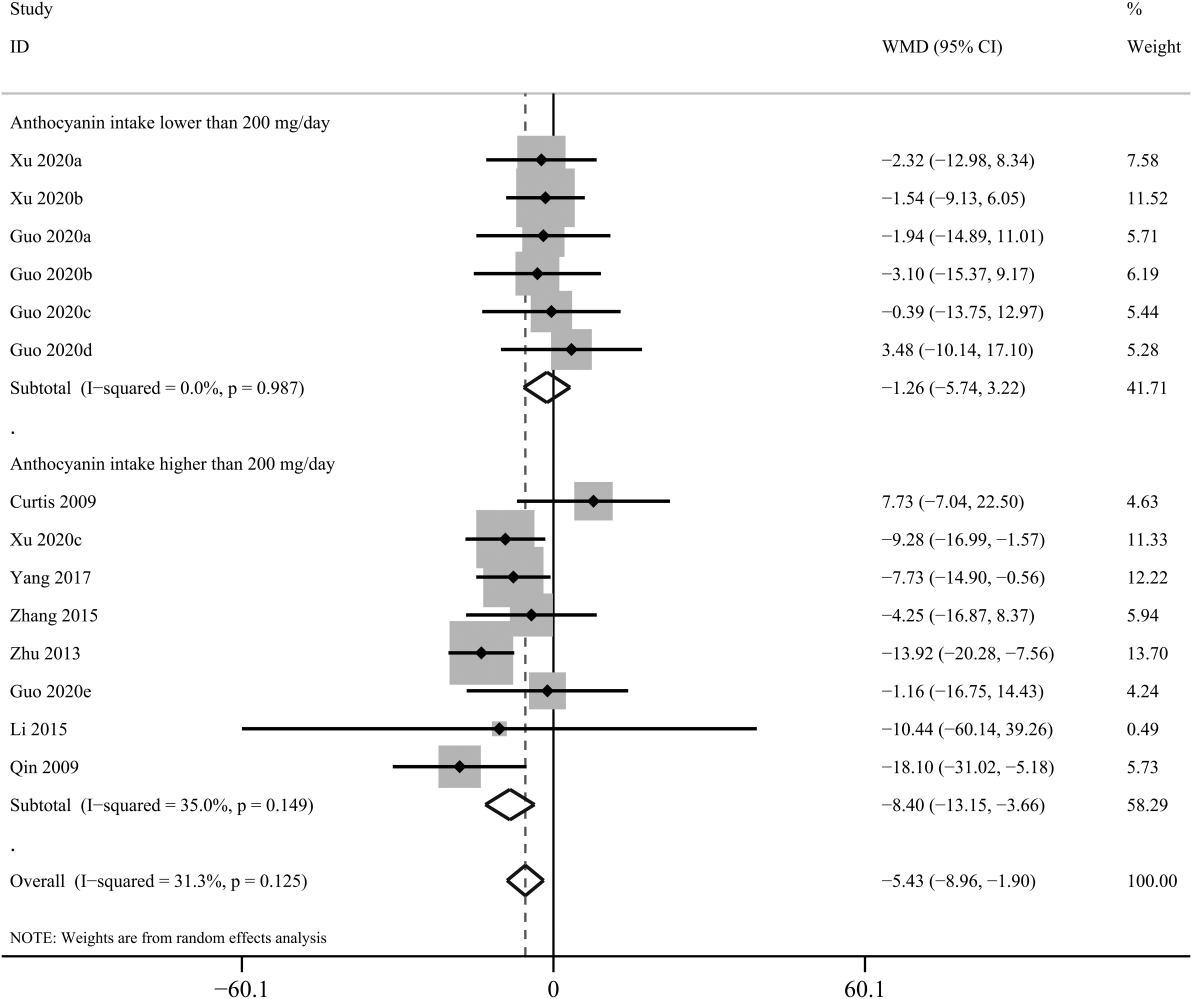
Forest plot for the pooled effects of purified anthocyanins on circulating low-density lipoprotein cholesterol stratified by anthocyanin doses. Between-study heterogeneity was examined using the Cochrane's Q test. The diamonds represented the pooled effect sizes which were calculated using the DerSimonian–Laird random-effects model. WMD, weighted mean difference.

The effects of purified anthocyanins and anthocyanin-rich berries on blood lipids were inconsistent. We found considerable reductions in blood LDL-C concentrations after regular intake of purified anthocyanins (WMD: −5.43 mg/dL, 95% CI: −8.96, −1.90 mg/dL; *p*_difference_ = 0.003; *I*^2^ = 31.3%; 14 comparisons; 891 subjects; Figure 1 and Table 1) but not of anthocyanin-rich berries (WMD: −3.34 mg/dL, 95% CI: −7.39, 0.71 mg/dL; *p*_difference_ = 0.106; *I*^2^ = 81.8%; 14 comparisons; 620 subjects). Additionally, the reductions in LDL-C were more obvious in subjects taking ≥200 mg/day purified anthocyanins (WMD: −8.40 mg/dL, 95% CI: −13.15, −3.66 mg/dL; *p*_difference_ = 0.001; *I*^2^ = 35.0%; eight comparisons; 670 subjects) and did not alter when we restricted studies to those without funding from industry (WMD: −6.25 mg/dL, 95% CI: −9.58, −2.93 mg/dL; *p*_difference_ < 0.001; *I*^2^ = 22.0%; 13 comparisons; 841 subjects). We did not analyze the effects of anthocyanin-rich berries stratified by anthocyanin doses as this information was not available in several studies (35–41).

**Table 1:** Pooled effects of purified anthocyanins and anthocyanin-rich berries on circulating LDL cholesterol.

**Variables**	**Number of comparisons** **(subjects)**	**WMD (95% CI),** **mg/dL**	***p*** **_difference_**	***I*** **^2^, %**	***p*** **_heterogeneity_**
*Purified anthocyanins*					
Overall	14 (891)	−5.43 (−8.96, −1.90)	0.003	31.3	0.125
**Study design**					
Parallel	14 (891)	−5.43 (−8.96, −1.90)	0.003	31.3	0.125
**Duration**					
<8 weeks	5 (107)	−0.73 (−6.74, 5.27)	0.811	0.0	0.969
≥8 weeks	9 (784)	−7.05 (−11.53, −2.58)	0.002	44.2	0.074
**Health status of subjects**					
Low CVD risk	5 (107)	−0.73 (−6.74, 5.27)	0.811	0.0	0.969
High CVD risk	9 (784)	−7.05 (−11.53, −2.58)	0.002	44.2	0.074
**Type of control**					
Placebo	14 (891)	−5.43 (−8.96, −1.90)	0.003	31.3	0.125
**Dose of anthocyanins**					
<200 mg/day	6 (221)	−1.26 (−5.74, 3.22)	0.582	0.0	0.987
≥200 mg/day	8 (670)	−8.40 (−13.15, −3.66)	0.001	35.0	0.149
**Study quality**					
Low to moderate	2 (178)	−17.62 (−30.12, −5.11)	0.006	0.0	0.770
High	12 (713)	−4.76 (−8.27, −1.25)	0.008	29.0	0.161
**Received industry funding?**					
Yes	1 (50)	7.73 (−7.04, 22.50)	0.305	–	–
No	13 (841)	−6.25 (−9.58, −2.93)	<0.001	22.0	0.221
*Anthocyanin-rich berries*					
Overall	14 (620)	−3.34 (−7.39, 0.71)	0.106	81.8	<0.001
**Type of berry**					
Blueberry	7 (309)	−0.97 (−6.43, 4.48)	0.727	88.2	<0.001
Cranberry	5 (230)	−5.38 (−18.47, 7.72)	0.421	72.7	0.005
Bilberry	2 (81)	−4.90 (−10.94, 1.15)	0.112	26.6	0.243
**Study design**					
Parallel	10 (438)	−3.81 (−8.59, 0.98)	0.119	87.3	<0.001
Crossover	4 (182)	−1.73 (−7.69, 4.22)	0.568	0.0	0.930
**Duration**					
<8 weeks	5 (214)	−1.66 (−7.37, 4.06)	0.570	0.0	0.978
≥8 weeks	9 (406)	−3.96 (−8.89, 0.98)	0.116	88.7	<0.001
**Health status of subjects**					
Low CVD risk	2 (86)	1.29 (−8.44, 11.03)	0.795	0.0	0.371
High CVD risk	12 (534)	−3.80 (−8.14, 0.54)	0.086	84.3	<0.001
**Type of control**					
Placebo	10 (476)	−3.13 (−7.73, 1.46)	0.181	86.6	<0.001
Others	4 (144)	−5.74 (−12.20, 0.73)	0.082	0.0	0.468
**Study quality**					
Low to moderate	6 (193)	−5.66 (−17.85, 6.54)	0.363	55.1	0.049
High	8 (427)	−1.98 (−6.30, 2.35)	0.371	87.2	<0.001
**Received industry funding?**					
Yes	10 (483)	−0.29 (−4.98, 4.40)	0.903	82.9	<0.001
No	4 (137)	−9.98 (−19.44, −0.53)	0.038	71.4	0.015
**Type of intervention**					
Powder	9 (394)	−4.13 (−8.84, 0.57)	0.085	88.4	<0.001
Beverage	4 (200)	0.97 (−6.67, 8.61)	0.803	0.0	0.638
Fresh fruits	1 (26)	−7.74 (−29.79, 14.31)	0.491	-	-

*CVD, cardiovascular disease; WMD, weighted mean difference*.

*The pooled effects sizes and 95% CIs were calculated using the random-effects model. Between-study heterogeneity was examined using the Cochrane's Q test*.

Besides, administration of purified anthocyanins could lead to substantial elevations in blood HDL-C (WMD: 11.49 mg/dL, 95% CI: 7.43, 15.55 mg/dL; *p*_difference_ < 0.001; *I*^2^ = 93.5%; 14 comparisons; 893 subjects; Supplementary Table 12) despite significant between-study heterogeneity. After excluding the study by Guo et al. (42), the effects on HDL-C were attenuated but remained statistically significant (WMD: 2.76 mg/dL, 95% CI: 1.34, 4.18 mg/dL; *p*_difference_ < 0.001; *I*^2^ = 43.5%; nine comparisons; 786 subjects). Subgroup analysis suggested that the effects on HDL-C were not significantly influenced by study duration, health status of subjects, anthocyanin doses, study quality, and funding source. In contrast, among anthocyanin-rich berries, only blueberry could slightly increase blood HDL-C concentrations (WMD: 1.46 mg/dL, 95% CI: 0.20, 2.72 mg/dL; *p*_difference_ = 0.023; *I*^2^ = 85.9%; seven comparisons; 309 subjects).

However, present meta-analysis showed that supplementation of purified anthocyanins did not significantly affect circulating concentrations of TC (WMD: −2.17 mg/dL, 95% CI: −5.74, 1.40 mg/dL; *p*_difference_ = 0.234; *I*^2^ = 0.0%; 16 comparisons; 975 subjects; Supplementary Table 13). The effects of purified anthocyanins on TC did not differ significantly when subgrouping by study characteristics. Nevertheless, administration of anthocyanin-rich berries could slightly but significantly reduce blood TC (WMD: −4.48 mg/dL, 95% CI: −8.94, −0.02 mg/dL; *p*_difference_ = 0.049; *I*^2^ = 86.4%; 20 comparisons; 895 subjects) particularly in subjects that were at high risks of developing CVDs (WMD: −6.09 mg/dL, 95% CI: −11.09, −1.08 mg/dL; *p*_difference_ = 0.017; *I*^2^ = 89.9%; 15 comparisons; 709 subjects).

**Figure 2:**
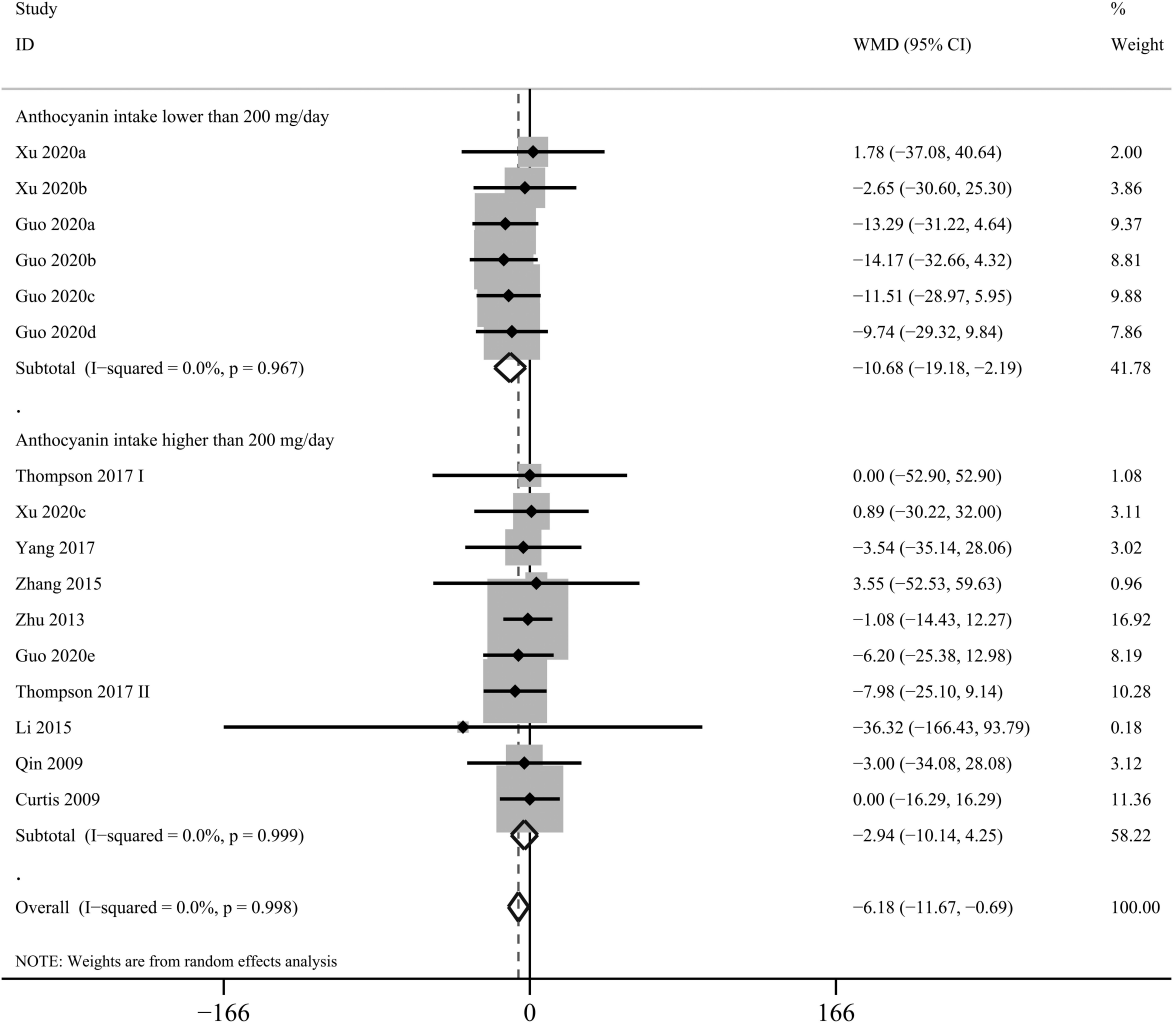
Forest plot for the pooled effects of purified anthocyanins on circulating triglyceride stratified by anthocyanin doses. Between-study heterogeneity was examined using the Cochrane's Q test. The diamonds represented the pooled effect sizes which were calculated using the DerSimonian–Laird random-effects model. WMD, weighted mean difference.

The effects of purified anthocyanins and anthocyanin-rich berries on blood TG had similar results with those on LDL-C. Rather than anthocyanin-rich berries (WMD: 6.02 mg/dL, 95% CI: −0.37, 12.40 mg/dL; *p*_difference_ = 0.065; *I*^2^ = 75.2%; 20 comparisons; 892 subjects; Table 2), we observed that purified anthocyanins could effectively decrease blood TG with only non-significant between-study heterogeneity detected (WMD: −6.18 mg/dL, 95% CI: −11.67, −0.69 mg/dL; *p*_difference_ = 0.027; *I*^2^ = 0%; 16 comparisons; 973 subjects; Figure 2).

**Table 2:** Pooled effects of purified anthocyanins and anthocyanin-rich berries on circulating triglyceride.

**Variables**	**Number of comparisons (subjects)**	**WMD (95% CI), mg/dL**	***p*** **_difference_**	***I*** **^2^, %**	***p*** **_heterogeneity_**
*Purified anthocyanins*					
Overall	16 (973)	−6.18 (−11.67, −0.69)	0.027	0.0	0.998
**Study design**					
Parallel	14 (891)	−6.05 (−11.88, −0.21)	0.042	0.0	0.994
Crossover	2 (82)	−7.22 (−23.51, 9.07)	0.385	0.0	0.778
**Duration**					
<8 weeks	7 (189)	−10.32 (−17.69, −2.95)	0.006	0.0	0.995
≥8 weeks	9 (784)	−1.02 (−9.25, 7.21)	0.809	0.0	1.000
**Health status of subjects**					
Low CVD risk	6 (139)	−10.53 (−17.97, −3.08)	0.006	0.0	0.991
High CVD risk	10 (834)	−0.99 (−9.12, 7.14)	0.811	0.0	1.000
**Type of control**					
Placebo	16 (973)	−6.18 (−11.67, −0.69)	0.027	0.0	0.998
**Dose of anthocyanins**					
<200 mg/day	6 (221)	−10.68 (−19.18, −2.19)	0.014	0.0	0.967
≥200 mg/day	10 (752)	−2.95 (−10.14, 4.25)	0.422	0.0	0.999
**Study quality**					
Low to moderate	2 (178)	−4.80 (−35.03, 25.43)	0.756	0.0	0.625
High	14 (795)	−6.23 (−11.81, −0.64)	0.029	0.0	0.995
**Received industry funding?**					
Yes	1 (50)	0.00 (−16.29, 16.29)	1.000	–	–
No	15 (923)	−6.97 (−12.80, −1.14)	0.019	0.0	0.999
*Anthocyanin-rich berries*					
Overall	20 (892)	6.02 (−0.37, 12.40)	0.065	75.2	<0.001
**Type of berry**					
Blueberry	8 (353)	9.06 (−0.87, 19.00)	0.074	85.4	<0.001
Cranberry	9 (396)	3.66 (−5.86, 13.18)	0.451	36.1	0.130
Bilberry	3 (143)	6.62 (−3.09, 16.32)	0.181	5.1	0.349
**Study design**					
Parallel	17 (736)	6.72 (−0.32, 13.76)	0.061	77.2	<0.001
Crossover	3 (156)	5.10 (−4.53, 14.73)	0.299	0.0	0.466
**Duration**					
<8 weeks	8 (340)	6.60 (−0.83, 14.03)	0.081	0.0	0.589
≥8 weeks	12 (552)	5.94 (−2.26, 14.13)	0.156	83.0	<0.001
**Health status of subjects**					
Low CVD risk	4 (148)	9.31 (−6.20, 24.81)	0.240	7.9	0.354
High CVD risk	16 (744)	5.50 (−1.44, 12.44)	0.120	79.4	<0.001
**Type of control**					
Placebo	14 (644)	6.94 (−0.17, 14.04)	0.056	80.9	<0.001
Others	6 (248)	3.33 (−8.78, 15.43)	0.590	0.0	0.511
**Study quality**					
Low to moderate	9 (317)	5.59 (−5.07, 16.24)	0.304	0.0	0.667
High	11 (575)	6.48 (−1.07, 14.03)	0.093	85.1	<0.001
**Received industry funding?**					
Yes	14 (651)	6.55 (−1.03, 14.12)	0.090	80.4	<0.001
No	6 (241)	6.24 (−2.02, 14.50)	0.139	0.0	0.464
**Type of intervention**					
Powder	11 (480)	4.80 (−4.11, 13.71)	0.291	83.0	<0.001
Beverage	8 (386)	6.51 (−2.22, 15.24)	0.144	34.0	0.157
Fresh fruits	1 (26)	16.83 (−8.14, 41.80)	0.186	–	–

*CVD, cardiovascular disease; WMD, weighted mean difference*.

*The pooled effects sizes and 95% CIs were calculated using the random-effects model. Between-study heterogeneity was examined using the Cochrane's Q test*.

### Pooled Effects of Anthocyanins and Anthocyanin-Rich Berries on Chronic Low-Grade Inflammation

Circulating CRP and TNF-α are two commonly used biomarkers of chronic low-grade inflammation. We found reduced blood concentrations of TNF-α due to supplementation of purified anthocyanins (WMD: −1.62 pg/mL, 95% CI: −2.76, −0.48 pg/mL; *p*_difference_ = 0.005; *I*^2^ = 0.0%; nine comparisons; 481 subjects; Supplementary Table 14) but not of anthocyanin-rich berries (WMD: 0.10 pg/mL, 95% CI: −0.15, 0.35 pg/mL; *p*_difference_ = 0.436; *I*^2^ = 0.0%; 10 comparisons; 460 subjects). In addition, treatment of either purified anthocyanins (WMD: −0.028 mg/dL, 95% CI: −0.050, −0.005 mg/dL; *p*_difference_ = 0.014; *I*^2^ = 26.0%; eight comparisons; 579 subjects) or anthocyanin-rich berries (WMD: −0.046 mg/dL, 95% CI: −0.070, −0.022 mg/dL; *p*_difference_ < 0.001; *I*^2^ = 0.0%; 13 comparisons; 655 subjects) significantly lowered circulating CRP as shown in Supplementary Table 15.

### Pooled Associations Between Anthocyanins and CHD Incidence and Mortality

**Figure 3:**
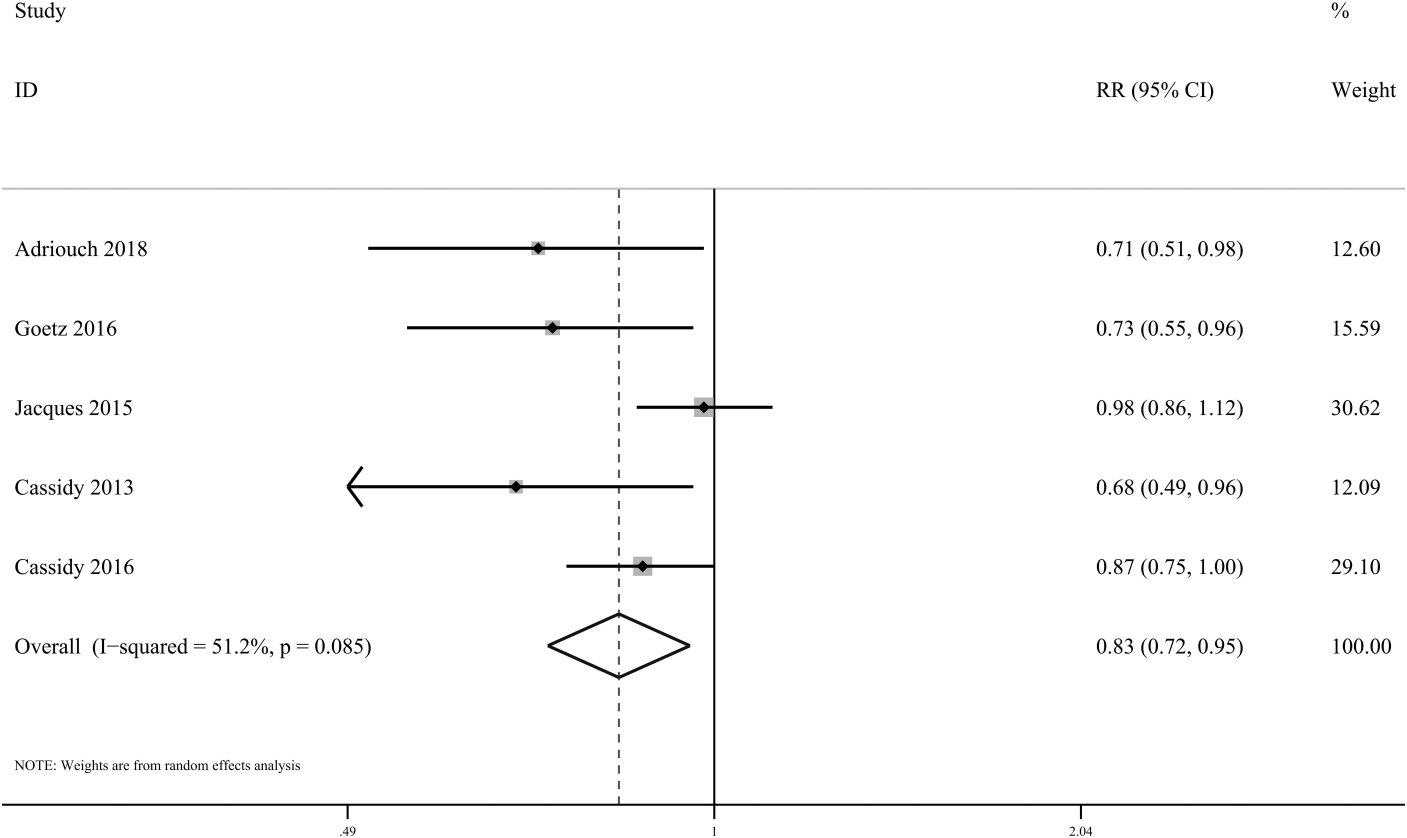
Forest plot for the pooled associations of dietary anthocyanins with incidence of CHD. Between-study heterogeneity was examined using the Cochrane's Q test. The diamond represented the pooled risk estimate which was calculated using the DerSimonian–Laird random-effects model. RR, relative risk.

Five eligible cohorts including 2,41,196 subjects and 3,786 cases evaluated the associations of dietary anthocyanin with CHD incidence. We found high anthocyanin intake was related to 17% lower incidence of CHD (*RR*: 0.83, 95% CI: 0.72, 0.95; *p*_difference_ = 0.009; *I*^2^ = 51.2%; Figure 3 and Supplementary Table 16). However, habitual consumption of anthocyanin was not related to reduced deaths from CHD (*RR*: 0.98, 95% CI: 0.79, 1.22; *p*_difference_ = 0.844; *I*^2^ = 80.1%; two cohorts, 78,369 subjects, 3,145 cases).

### Pooled Associations Between Anthocyanins and Stroke Incidence and Mortality

In the present meta-analysis, we found that dietary anthocyanins were not associated with incidence of total stroke (*RR*: 0.84, 95% CI: 0.62, 1.14; *p*_difference_ = 0.256; *I*^2^ = 91.0%; three cohorts, 19,766 subjects, 3,668 cases; Supplementary Table 17), ischemic stroke (*RR*: 0.91, 95% CI: 0.78, 1.05; *p*_difference_ = 0.202; *I*^2^ = 0.0%; three cohorts, 1,15,452 subjects, 1,997 cases; Supplementary Table 18), or hemorrhagic cases) or total stroke mortality (*RR*: 1.01, 95% CI: 0.83, 1.24; *p*_difference_ = 0.923; one cohort, 34,489 subjects, 469 cases).

### Pooled Associations Between Anthocyanins and Total CVD Incidence and Mortality

**Figure 4:**
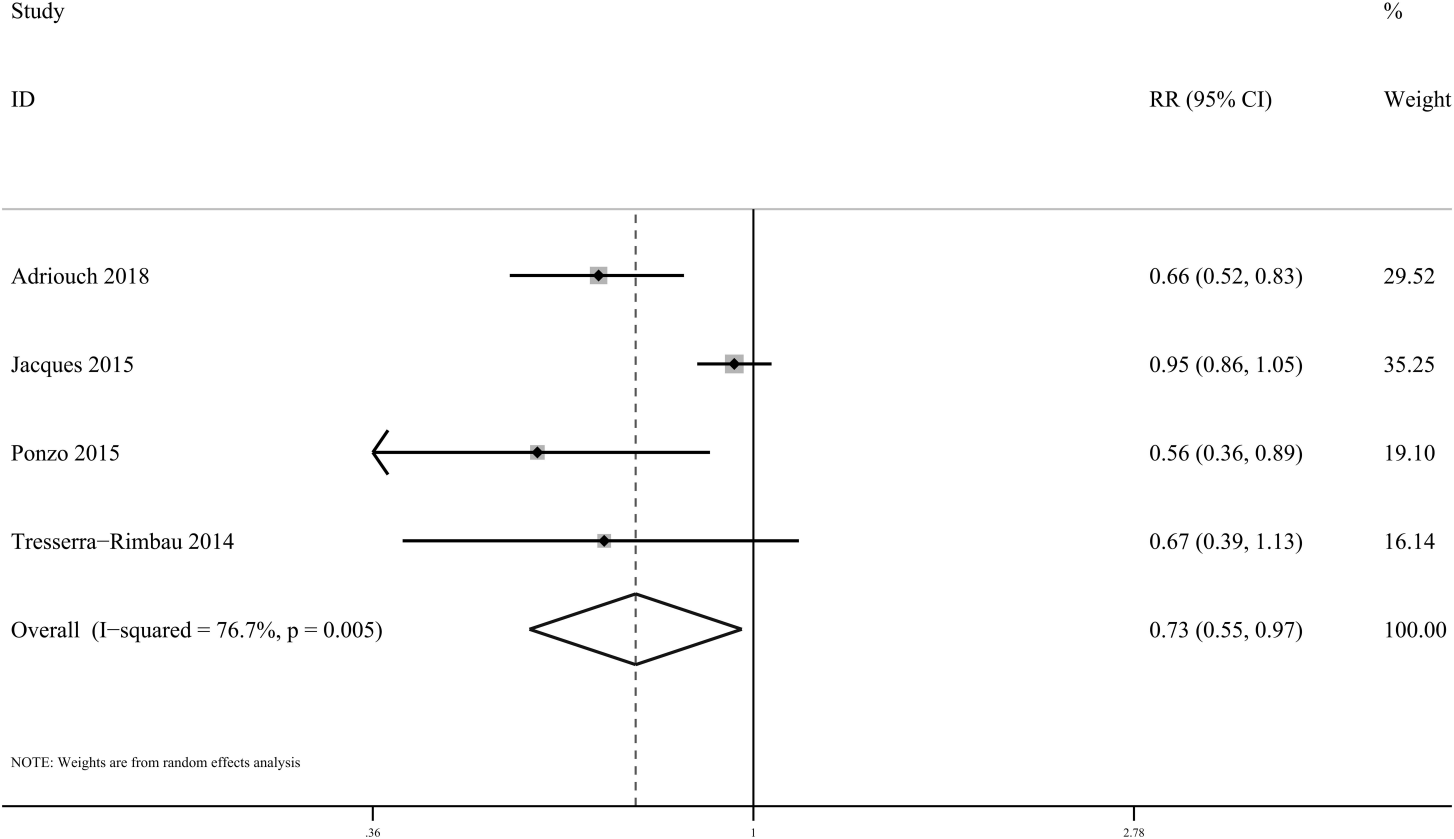
Forest plot for the pooled associations of dietary anthocyanins with incidence of total CVDs. Between-study heterogeneity was examined using the Cochrane's Q test. The diamond represented the pooled risk estimate which was calculated using the DerSimonian–Laird random-effects model. RR, relative risk.

**Figure 5:**
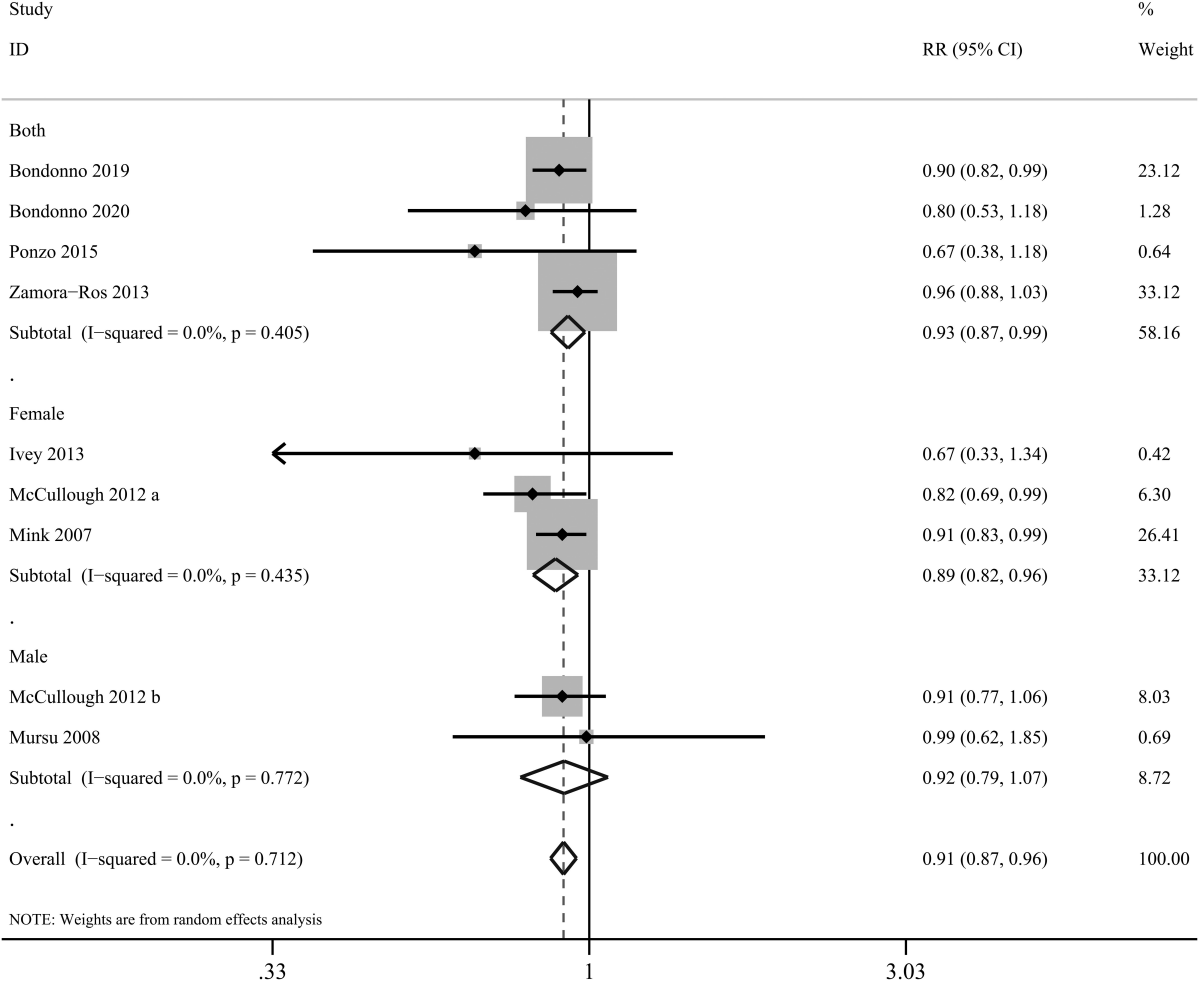
Forest plot for the pooled associations of dietary anthocyanins with mortality from total CVDs. Between-study heterogeneity was examined using the Cochrane's Q test. The diamonds represented the pooled risk estimates which were calculated using the DerSimonian–Laird random-effects model. RR, relative risk.

In the present meta-analysis, anthocyanin intake was linked to 27% lower risk of total CVDs (*RR*: 0.73, 95% CI: 0.55, 0.97; *p*_difference_ = 0.030; *I*^2^ = 76.7%; four cohorts, 95,868 subjects, 1,518 cases; Figure 4 and Supplementary Table 19). Besides, there was significant inverse relationship between dietary anthocyanins and mortality from total CVDs (*RR*: 0.91, 95% CI: 0.87, 0.96; *p*_difference_ < 0.001; *I*^2^ = 0.0%; nine cohorts, 2,36,648 subjects, 9,765 cases; Figure 5 and Supplementary Table 19). Subgroup analysis revealed that the protective roles of dietary anthocyanins against total CVD mortality might be more obvious in women (*RR*: 0.89, 95% CI: 0.82, 0.96; *p*_difference_ = 0.003; *I*^2^ = 0.0%; three cohorts, 95,841 subjects, 3,562 cases) than in men (*RR*: 0.92, 95% CI: 0.79, 1.07; *p*_difference_ = 0.263; *I*^2^ = 0.0%; two cohorts, 40,130 subjects, 1,419 cases).

### Publication Bias

No significant systematic publication bias was found for each outcome except those reporting the effects of purified anthocyanins on HDL-C (Begg's *p* = 0.016). The trim and fill method did not satisfactorily correct the theoretically unpublished or missing studies. Alternatively, after we excluded the study by Guo et al. (42), significance of the Begg's test for publication bias turned into null (Begg's *p* = 0.754).

## Discussion

In the present meta-analysis of RCTs and prospective cohort studies, we demonstrated that administration of purified anthocyanins effectively improved blood lipid profiles and reduced circulating CRP and TNF-α, biomarkers of chronic low-grade inflammation, while not affecting adiposity, blood pressure, or FMD. Supplementation of anthocyanin-rich berries could also moderately decrease blood concentrations of TC and CRP, albeit the ameliorative effects were less remarkable than those of purified anthocyanins. We also found that high dietary intake of anthocyanins was associated with lower CHD risk and also total CVD incidence and mortality in the pooled analysis of prospective cohort studies.

The blood lipid modulatory effects of anthocyanins have been well documented in humans and experimental animals before (20, 23, 43, 44). Specifically, anthocyanin supplementation could inhibit cholesteryl ester transfer protein (CETP) leading to lower circulating proatherogenic LDL-C but raised antiatherogenic HDL-C in the dyslipidemia (45). In the present meta-analysis, we further showed that the reductions in LDL-C were only statistically significant in subjects consuming ≥200 mg/day of anthocyanins, whereas the regulatory effects on HDL-C and TG were more obvious in those received <200 mg anthocyanin per day. In contrast to present findings, our previous study suggested significant linear trends for the dose-related effects of anthocyanins on HDL-C but not on LDL-C (46). It is possible that other confounding factors including adherence to intervention and baseline health status of subjects might substantially influence the blood lipid modulatory properties of dietary anthocyanins. Besides, anthocyanins might affect lipid metabolism *via* alternative molecular pathways other than CETP. Therefore, future studies are warranted to disentangle the dose-related effects of anthocyanin intake on blood lipids.

Notwithstanding decreased circulating proinflammatory CRP in response to either purified anthocyanins or anthocyanin-rich berries in the present meta-analysis of RCTs, the antiinflammatory benefits should be interpretated with caution as the cut points for prognostic usages of CRP are still lacking. Besides, it remained unclear whether the observed antiinflammatory properties of anthocyanins and anthocyanin-rich berries were directly from themselves or just intermediate effects.

Berries are the most important dietary sources of anthocyanins (11). Even though all anthocyanin supplements that used in the included RCTs were produced from berries, the cardiovascular benefits of purified anthocyanins seemed more remarkable compared with those of anthocyanin-rich berries in this study. However, the incompleteness of data to estimate the daily anthocyanin intake from berries (35–41) along with the varying intervention approaches in the included anthocyanin-rich berry studies made it arbitrary, at least now, to draw a conclusion about the difference between anthocyanin-rich berries and purified anthocyanins. Moreover, in addition to anthocyanins, berries also contain abundant soluble fibers, manganese, vitamins C and K, and other polyphenols (47, 48). Administration of berry fruits could enhance glycemic control, urinary tract health, and cognitive function beyond their cardioprotective effects (26, 27). Thus, we suggested that the inferior hypolipidemic and anti-inflammatory efficacies of anthocyanin-rich berries to purified anthocyanin supplements observed in the present meta-analysis should not neglect the health-promoting roles of berries.

Although the outcomes of interests in the present meta-analysis of RCTs were surrogate markers of CVDs rather than CVD events, our results were of clinical relevance for CVD prevention and treatment. Earlier Mendelian randomization analyses suggested that per mmol/L (38.7 mg/dL) decrement in blood LDL-C and HDL-C was associated with 54.5% lower and 47.0% higher risks of CHD, respectively (49, 50). Accordingly, 11.49 mg/dL increment in HDL-C and 5.43 mg/dL reduction in LDL-C due to purified anthocyanin supplementation might associate with 14.0 and 7.6% lower incidence of CHD, respectively. Consistently, our pooled analysis of prospective cohort studies further showed that regular anthocyanin consumption was related to 17, 27, and 9% lower risk of CHD risk, total CVD incidence, and total CVD deaths, respectively.

Compared with two previous meta-analyses (20, 23), one strength of this study was that we separately evaluated the effects of purified anthocyanins and anthocyanin-rich berries. As a result, we observed only minor between-study heterogeneity in purified anthocyanin studies in most outcomes. However, the heterogeneity among berry studies remained high for most outcomes while stratifying by study characteristics did not convincingly solve the source of heterogeneity. It is possible that the age of participants, bioavailability and doses of different anthocyanin species, or other confounding factors lead to the observed inconsistence among studies. Particularly, in subjects with obesity, dyslipidemia, diabetes, or past or present CVDs, the comorbidity might influence the cardioprotective efficacy of anthocyanins and anthocyanin-rich berries. Recent studies have highlighted the involvement of gut microbiota in individual-specific response to phytochemicals (51). Due to their low bioavailability, the cardioprotective benefits of anthocyanins have been proven to partly depend on gut microbiota (52, 53). Therefore, unraveling the person-specific interactions between dietary anthocyanins and gut microbiota might help to address the heterogeneous physiological responses due to dietary anthocyanins and anthocyanin-rich berries among subjects.

Limitations of the present meta-analysis should be put forward. First, most of the RCTs included in the present meta-analysis were of relatively small sizes and short durations. However, the total sample size of the included RCTs was about two-fold larger than those in two previous meta-analyses of RCTs concerning the effects of anthocyanins on cardiometabolic health (20, 23). Second, we only focused on major anthocyanin-rich berries that were frequently consumed in this study. Potential cardiovascular benefits of other berry species that are less popular need future investigations. Third, about half of the included RCTs obtained financial supports from berry industry or industry association which might lead to selective reporting of positive results (54). Nevertheless, subgrouping by funding source did not find any more benefits of purified anthocyanins or anthocyanin-rich berries on each surrogate marker of CVDs in the present meta-analysis. Besides, we observed significant between-study heterogeneity even after subgroup analysis stratified by various study characteristics. Future well-designed clinical trials are warranted to clarify the source of heterogeneity.

In conclusion, this study updated and extended current clinical and epidemiological evidence about the protective roles of purified anthocyanins and anthocyanin-rich berries on cardiovascular health. Our results suggested that regular consumption of either purified anthocyanins or anthocyanin-rich berries could prevent CVDs through their lipid-lowering and anti-inflammatory properties. We also propose that anthocyanins and anthocyanin-rich berries should be taken into consideration when formulating cardioprotective diets in the future.

## Data Availability Statement

The original contributions presented in the study are included in the article/Supplementary Material, further inquiries can be directed to the corresponding author/s.

## Author Contributions

YY and YZ designed research. LX, HC, ZT, and YZ conducted research. LX and HC performed statistical analysis. LX and YZ wrote paper. YY and YZ had primary responsibility for final content. All authors have read and approved the final manuscript.

## Funding

This work was supported by the National Natural Science Foundation of China (Grant Numbers 81730090 and 81973022), Guangzhou Science, Technology and Innovation Committee (Grant Number 201804020045), Guangdong Basic and Applied Basic Research Foundation (Grant Number 2019A1515111103), and CNS Research Fund for DRI.

## Conflict of Interest

The authors declare that the research was conducted in the absence of any commercial or financial relationships that could be construed as a potential conflict of interest.

## Publisher's Note

All claims expressed in this article are solely those of the authors and do not necessarily represent those of their affiliated organizations, or those of the publisher, the editors and the reviewers. Any product that may be evaluated in this article, or claim that may be made by its manufacturer, is not guaranteed or endorsed by the publisher.

## Abbreviations

CVD: cardiovascular disease
CETP: cholesteryl ester transfer protein
CI: confidence interval
CHD: coronary heart disease
CRP: C-reactive protein
DBP: diastolic blood pressure
FMD: flow-mediated dilation
FFQ: food frequency questionnaire
HR: hazard ratio
HDL-C: high-density lipoprotein cholesterol
PROSPERO: International Prospective Register of Systematic Reviews
LDL-C: low-density lipoprotein cholesterol
NHLBI: National Heart, Lung, and Blood Institute
PRISMA: Preferred Reporting Items for Systematic Reviews and Meta-Analyses
RCT: randomized controlled trial
RR: relative risk
SD: standard deviation
SBP: systolic blood pressure
TC: total cholesterol
TG: triglyceride
TNF-α: tumor necrosis factor alpha
WMD: weighted mean difference.
