# Supplementary material for: Anthocyanins, Anthocyanin-Rich Berries, and Cardiovascular Risks: Systematic Review and Meta-Analysis of 44 Randomized Controlled Trials and 15 Prospective Cohort Studies
Source: Front Nutr. 2021 Dec 15;8:747884. doi: 10.3389/fnut.2021.747884 (PMC8714924; doi:10.3389/fnut.2021.747884)
Supplement: Supplementary file 1 [file Data_Sheet_1.docx]

***Supplemental Material***

1. **Supplemental literature search strategy for randomized controlled trials and prospective cohort studies**
2. **Figure S1** Flow chart for literature search and identification of randomized controlled trials.
3. **Figure S2** Flow chart for literature search and identification of prospective cohort studies.
4. **Table S1** Anthocyanin contents in berries and other anthocyanin-rich fruits.
5. **Table S2** Characteristics of included randomized controlled trials.
6. **Table S3** Characteristics of included prospective cohort studies**.**
7. **Table S4** Summary of quality assessment for included randomized controlled trials**.**
8. **Table S5** Detailed results of quality assessment of included randomized controlled trials.
9. **Table S6** Summary of quality assessment for included prospective cohort studies.
10. **Table S7** Summary of quality assessment for included prospective cohort studies
11. **Table S8** Pooled effects of purified anthocyanins and anthocyanin-rich berries on BMI.
12. **Table S9** Pooled effects of purified anthocyanins and anthocyanin-rich berries on systolic blood pressure.
13. **Table S10** Pooled effects of purified anthocyanins and anthocyanin-rich berries on diastolic blood pressure.
14. **Table S11** Pooled effects of purified anthocyanins and anthocyanin-rich berries on flow-mediated dilation.
15. **Table S12** Pooled effects of purified anthocyanins and anthocyanin-rich berries on circulating HDL cholesterol.
16. **Table S13** Pooled effects of purified anthocyanins and anthocyanin-rich berries on circulating total cholesterol.
17. **Table S14** Pooled effects of purified anthocyanins and anthocyanin-rich berries on circulating tumor necrosis factor alpha.
18. **Table S15** Pooled effects of purified anthocyanins and anthocyanin-rich berries on circulating C-reactive protein.
19. **Table S16** Pooled associations of anthocyanins with incidence and mortality of coronary heart disease.
20. **Table S17** Pooled associations of anthocyanins with incidence and mortality of total stroke.
21. **Table S18** Pooled associations of anthocyanins with incidence of ischemic and hemorrhagic stroke.
22. **Table S19** Pooled associations of anthocyanins with incidence and mortality of total cardiovascular diseases.
23. **Supplemental references**
24. **Supplemental literature search strategy for randomized controlled trials and prospective cohort studies**

*RCTs*

a) PubMed

- #1 (((((((blueberry) OR (cranberry)) OR (blackcurrant)) OR (bilberry)) OR (anthocyanin)) OR (anthocyanidin)) OR (cyanidin)) OR (delphinidin)
- #2 (((((((((((((((((((((((((cardiovascular) OR (TNF-α)) OR (tumor necrosis factor-α)) OR (CRP)) OR (C-reactive protein)) OR (LDL-C)) OR (low density lipoprotein-cholesterol)) OR (HDL-C)) OR (high density lipoprotein-cholesterol)) OR (TC)) OR (total cholesterol)) OR (TG)) OR (triglyceride)) OR (cholesterol)) OR (BMI)) OR (body mass index)) OR (SBP)) OR (systolic blood pressure)) OR (systolic pressure)) OR (DBP)) OR (diastolic blood pressure)) OR (diastolic pressure)) OR (blood pressure)) OR (FMD)) OR (flow-mediated dilation)) OR (endothelial function)
- #1 AND #2

b) Embase

- #1 'blueberry'/exp OR blueberry OR 'cranberry'/exp OR cranberry OR 'blackcurrant'/exp OR blackcurrant OR 'bilberry'/exp OR bilberry OR 'anthocyanin'/exp OR anthocyanin OR 'anthocyanidin'/exp OR anthocyanidin OR 'cyanidin'/exp OR cyanidin OR 'delphinidin'/exp OR delphinidin
- #2 ((((((((((((('cardiovascular'/exp OR cardiovascular OR 'tnf α' OR 'tumor'/exp OR tumor) AND ('necrosis'/exp OR necrosis) AND 'factor α' OR 'crp'/exp OR crp OR 'c reactive') AND ('protein'/exp OR protein) OR 'ldl c' OR low) AND ('density'/exp OR density) AND ('lipoprotein cholesterol'/exp OR 'lipoprotein cholesterol') OR 'hdl c' OR high) AND ('density'/exp OR density) AND ('lipoprotein cholesterol'/exp OR 'lipoprotein cholesterol') OR tc OR 'total'/exp OR total) AND ('cholesterol'/exp OR cholesterol) OR tg OR 'triglyceride'/exp OR triglyceride OR 'cholesterol'/exp OR cholesterol OR 'bmi'/exp OR bmi OR 'body'/exp OR body) AND ('mass'/exp OR mass) AND ('index'/exp OR index) OR sbp OR systolic) AND ('blood'/exp OR blood) AND ('pressure'/exp OR pressure) OR systolic) AND ('pressure'/exp OR pressure) OR 'dbp'/exp OR dbp OR diastolic) AND ('blood'/exp OR blood) AND ('pressure'/exp OR pressure) OR diastolic) AND ('pressure'/exp OR pressure) OR 'blood'/exp OR blood) AND ('pressure'/exp OR pressure) OR fmd OR 'flow mediated') AND dilation OR endothelial) AND ('function'/exp OR function)
- #1 AND #2

c) Cochrane Library

- #1 (blueberry OR cranberry OR blackcurrant OR bilberry OR anthocyanin OR anthocyanidin OR cyanidin OR delphinidin) (Word variations have been searched)
- #2 (cardiovascular OR TNF-α OR tumor necrosis factor-α OR CRP OR C-reactive protein OR LDL-C OR low density lipoprotein-cholesterol OR HDL-C OR high density lipoprotein-cholesterol OR TC OR total cholesterol OR TG OR triglyceride OR cholesterol OR BMI OR body mass index OR SBP OR systolic blood pressure OR systolic pressure OR DBP OR diastolic blood pressure OR diastolic pressure OR blood pressure OR FMD OR flow-mediated dilation OR endothelial function) (Word variations have been searched)
- #1 AND #2

*Prospective cohort studies*

a) PubMed

- #1 ((((((delphinidin) OR (cyanidin)) OR (anthocyanidin)) OR (anthocyanin)) OR (flavonoid)) OR (berry)) OR (berries)
- #2 ((((((((((((((heart disease) OR (chd)) OR (cardiovascular disease)) OR (cvd)) OR (coronary heart disease)) OR (myocardial infarction)) OR (MI)) OR (stroke)) OR (death)) OR (mortality)) OR (intracerebral hemorrhage)) OR (cerebral Infarction)) OR (hemorrhagic stroke)) OR (ischemic stroke)) OR (subarachnoid hemorrhage)
- #3 (((prospective) OR (cohort)) OR (observational)) OR (longitudinal)
- #1 AND #2 AND #3

b) Embase

- #1 'delphinidin'/exp OR delphinidin OR 'cyanidin'/exp OR cyanidin OR 'anthocyanidin'/exp OR anthocyanidin OR 'anthocyanin'/exp OR anthocyanin OR 'flavonoid'/exp OR flavonoid OR 'berry'/exp OR berry OR 'berries'/exp OR berries
- #2 'heart disease' OR chd OR 'cardiovascular disease' OR cvd OR 'ischemic heart disease' OR 'heart infarction' OR mi OR 'cerebrovascular accident' OR 'death' OR 'mortality' OR 'brain infarction' OR 'brain hemorrhage' OR 'brain ischemia' OR 'subarachnoid hemorrhage'
- #3 'prospective study' OR 'cohort analysis' OR 'observational study' OR 'longitudinal study'
- #1 AND #2 AND #3

c) Cochrane Library

- #1 delphinidin OR cyanidin OR anthocyanidin OR anthocyanin OR flavonoid OR berry OR berries
- #2 "heart-disease" OR chd OR cardiovascular disease OR CVD OR coronary heart disease OR myocardial infarction OR mi OR stroke OR death OR mortality OR intracerebral hemorrhage OR cerebral Infarction OR hemorrhagic stroke OR ischemic stroke OR subarachnoid hemorrhage
- #3 prospective OR cohort OR observational OR longitudinal
- #1 AND #2 AND #3

1. **Figure S1** Flow chart for literature search and identification of randomized controlled trials.


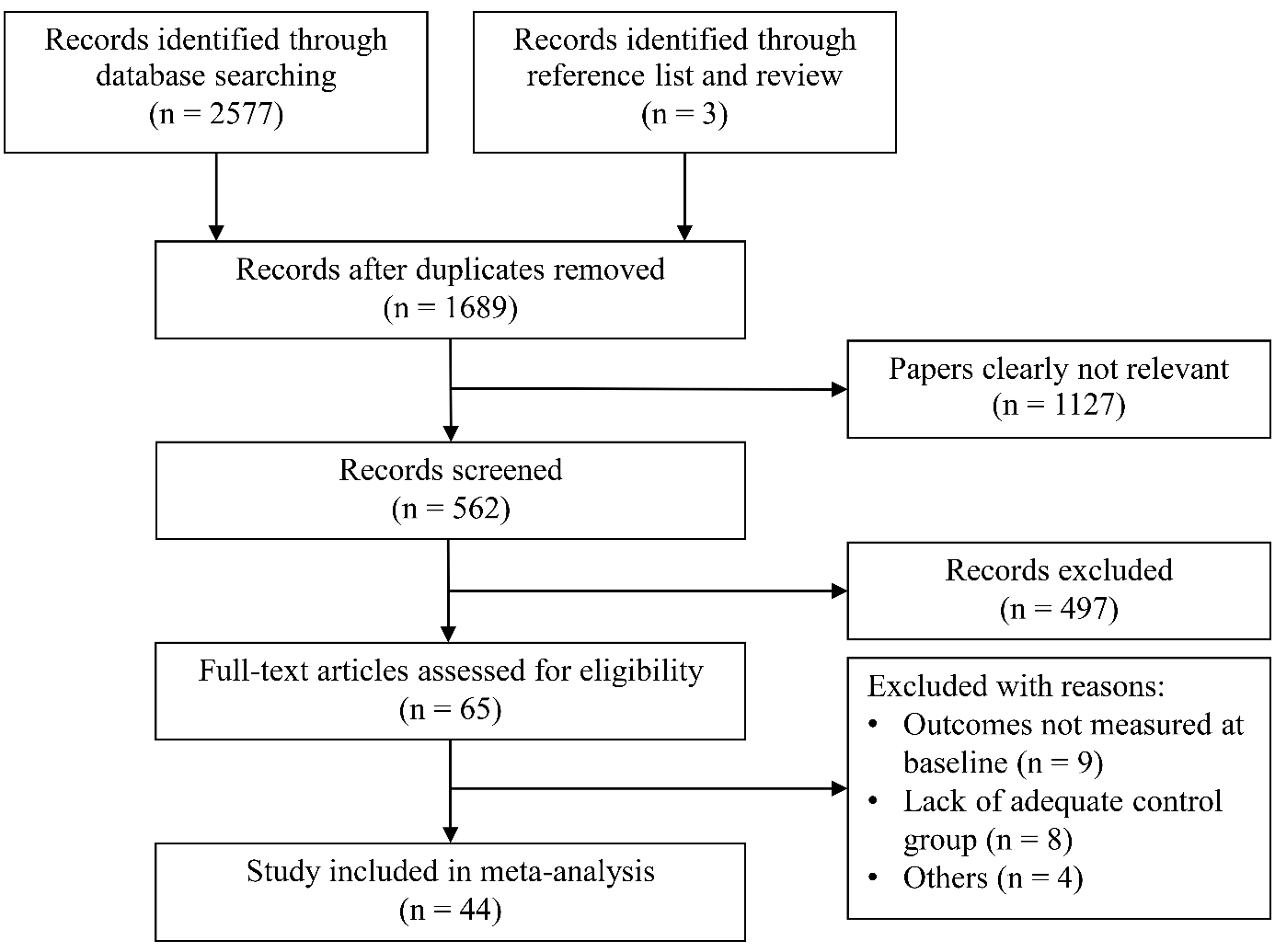


1. **Figure S2** Flow chart for literature search and identification of prospective cohort studies.


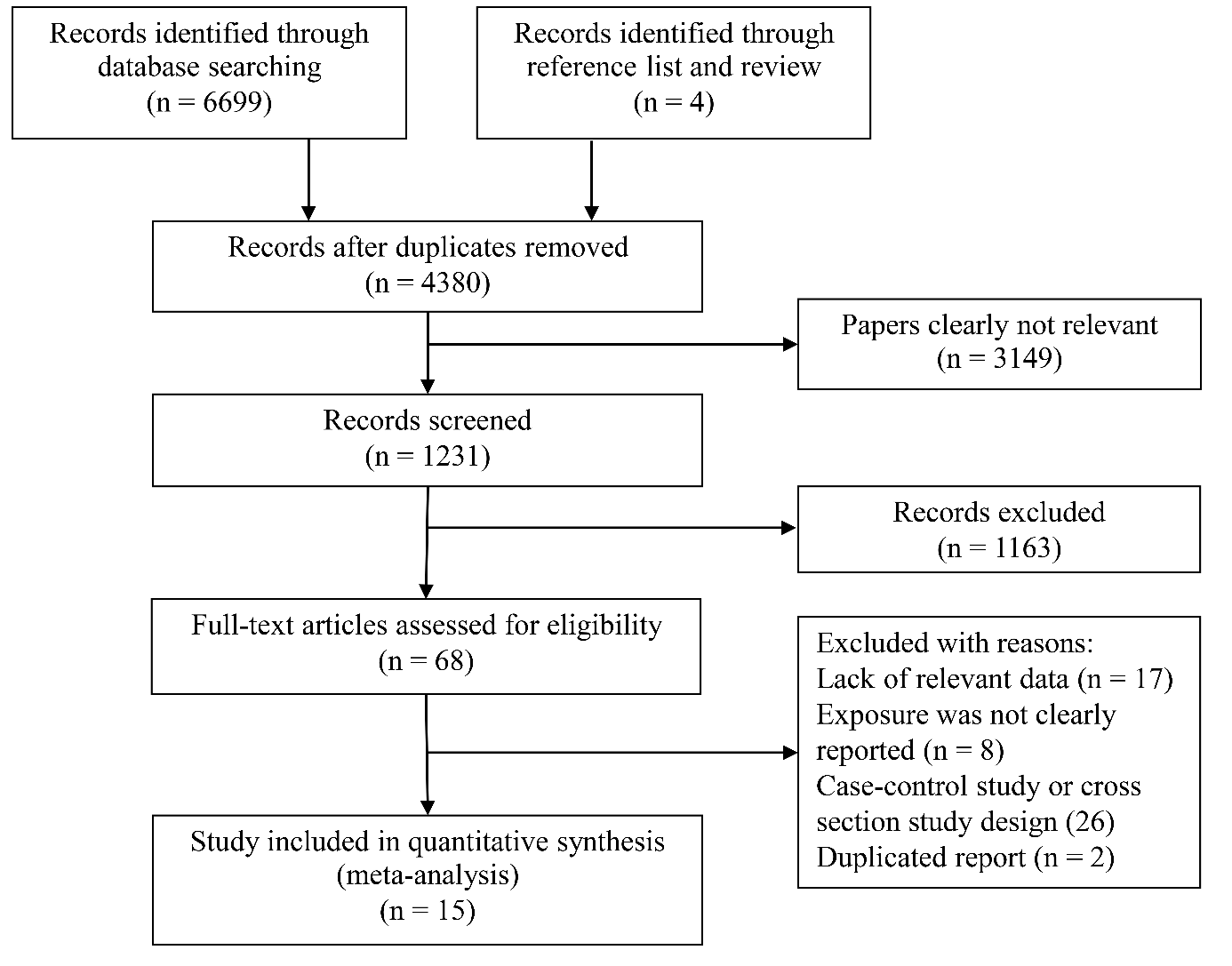


1. **Table S1** Anthocyanin contents in berries and other anthocyanin-rich fruits.^1, 2, 3^

| Fruits | Anthocyanin contents (mg/100g edible portions) | | | | | | |
| --- | --- | --- | --- | --- | --- | --- | --- |
|  | Cyanidin | Delphinidin | Malvidin | Pelargonidin | Peonidin | Petunidin | Total |
| Blueberry | 8.46 ± 1.79 | 35.43 ± 3.32 | 67.59 ± 3.50 | NA | 20.29 ± 4.43 | 31.53 ± 1.64 | 163.30 |
| Bilberry | 85.26 ± 4.84 | 97.59 ± 5.05 | 39.22 ± 1.70 | NA | 20.45 ± 1.79 | 42.69 ± 1.51 | 285.21 |
| Blackcurrant | 62.46 ± 60.1 | 89.62 ± 3.10 | NA | 1.17 ± 0.12 | 0.66 ± 0.11 | 3.87 ± 1.55 | 157.78 |
| Cranberry | 46.43 ± 7.95 | 7.67 ± 1.92 | 0.44 ± 0.31 | 0.32 ± 0.15 | 49.16 ± 8.07 | NA | 104.02 |
| Blackberry | 99.95 ± 6.96 | NA | NA | 0.45 ± 0.25 | 0.21 ± 0.21 | NA | 100.61 |
| Plum | 56.02 ± 22.88 | 0.01 | NA | 0.01 | NA | NA | 56.03 |
| Raspberry | 45.77 ± 6.74 | 1.32 ± 1.14 | 0.13 ± 0.13 | 0.98 ± 0.34 | 0.12 ± 0.12 | 0.31 ± 0.31 | 48.63 |
| Grape | 1.16 ± 0.36 | 2.27 ± 0.37 | 39 ± 6.83 | 0.02 | 3.62 ± 0.88 | 1.97 ± 0.33 | 48.04 |
| Strawberry | 1.68 ± 0.06 | 0.31 ± 0.29 | 0.01 ± 0.01 | 24.85 ± 0.70 | 0.05 ± 0.05 | 0.11 ± 0.11 | 27.01 |

^1^Abbreviations: NA, not available.

^2^Modified from the United States Department of Agriculture Database for the Flavonoid Content of Selected Foods Release 3.1 (2018).

^3^Data were expressed as means ± SDs.

1. **Table S2** Characteristics of included randomized controlled trials.^1^

| Study/country | Design/duration | Intervention | Anthocyanin intake, mg/day | Control | Gender (F/M) and health status of subjects | Outcomes | Received industry funding? |
| --- | --- | --- | --- | --- | --- | --- | --- |
| Alnajjar 2019/UK (Alnajjar et al., 2019) | C/3 wk | Bilberry, freeze-dried powder, 1.41 g/day | 507.6 | Placebo | 6/10, overweight | TG, TC, HDL-C, LDL-C, TNF-α | No |
| Arevström 2019/Sweden (Arevström et al., 2019) | P/8 wk | Bilberry, freeze-dried powder, 40 g/day | 900 | Others | 8/42, undergone myocardial infarction | BMI, SBP, DBP, TG, TC, HDL-C, LDL-C | No |
| Basu 2010/US (Basu et al., 2010) | P/8 wk | Blueberry, freeze-dried powder, 50 g/day | 742 | Others | 44/4, MetS patients | SBP, DBP, TG, TC, HDL-C, LDL-C, CRP | Yes |
| Basu 2011/US (Basu et al., 2011) | P/8 wk | Cranberry, beverage, 480 mL/day | 24.8 | Placebo | F:31, MetS patients | SBP, DBP, TG, TC, HDL-C, LDL-C, CRP | Yes |
| Chew 2019/US (Chew et al., 2019) | P/8 wk | Cranberry, beverage, 450 mL/day | 6.22 | Placebo | 45/33, overweight and obese | CRP, TNF-α | Yes |
| Curtis 2009/UK (Curtis et al., 2009) | P/12 wk | Anthocyanin capsules | 500 | Placebo | F: 52, postmenopausal females | BMI, SBP, DBP, TG, TC, HDL-C, LDL-C. CRP, TNF-α | Yes |
| Curtis 2019/UK (Curtis et al., 2019) | P/6 mon | Blueberry, freeze-dried powder, 13/26 g/day | 182/364 | Placebo | 37/78, MetS patients | SBP, DBP, TG, TC, HDL-C, LDL-C. FMD | Yes |
| Dohadwala 2011/US (Dohadwala et al., 2011) | C/4 wk | Cranberry, beverage, 480 mL/day | 94 | Placebo | 14/30, with coronary artery disease | SBP, DBP, TG, TC, HDL-C, LDL-C, FMD, CRP | Yes |
| Du 2019/US (Du et al., 2019) | P/4 mon | Blueberry, freeze-dried powder, 40 g/day | NA | Placebo | 47/16, osteoarthritis patients | BMI, SBP, DBP, TNF-α | Yes |
| Duthie 2006/UK (Duthie et al., 2006) | P/2 wk | Cranberry, beverage, 750 mL/day | NA | Placebo | F:20, healthy | TC, TG | Yes |
| Flammer 2013/US (Flammer et al., 2013) | P/4 mon | Cranberry, beverage, 460 mL/day | 69.46 | Placebo | 53/31, with peripheral endothelial dysfunction and cardiovascular risk factors | SBP, DBP, TG, TC, TNF-α | Yes |
| Guo 2020/China (Guo et al., 2020) | P/14 day | Anthocyanin capsules | 20/40/80/160/320 | Placebo | 72/39, healthy | BMI, SBP, DBP, TG, TC, HDL-C, LDL-C, TNF-α | No |
| Hassellund 2012/Norway (Hassellund et al., 2012) | C/4 wk | Anthocyanin capsules | 640 | Placebo | M:27, hypertension | SBP, DBP | Yes |
| Hsia 2020/US (Hsia et al., 2020) | P/8 wk | Cranberry, beverage, 450 mL/day | 6.5 | Placebo | 23/12, obese and hyperglycemia | SBP, DBP | Yes |
| Johnson 2015/US (Johnson et al., 2015) | P/8 wk | Blueberry, freeze-dried powder, 22 g/day | 469 | Placebo | W:40, postmenopausal females with pre- and stage 1-hypertension | BMI, SBP, DBP, CRP | Yes |
| Johnson 2017/US (Johnson et al., 2017) | P/8 wk | Blueberry, freeze-dried powder, 22 g/day | 469 | Placebo | W:40, postmenopausal females with pre- and stage 1-hypertension | TNF-α | Yes |
| Karlsen 2007/Norway (Karlsen et al., 2007) | P/3 wk | Anthocyanin capsules | 300 | Placebo | 58/60, healthy | CRP, TNF-α | No |
| Karlsen 2010/Norway (Karlsen et al., 2010) | P/4 wk | Bilberry, beverage, 330 mL/day | 359 | Others | 17/46, at elevated risk of CVD | TG, TC, CRP, TNF-α | No |
| Khan 2014/UK (Khan et al., 2014) | P/6 wk | Blackcurrant, beverage, 1000 mL/day | 40/143 | Placebo | 21/43, healthy but with low intake of fruit and vegetables | BMI, SBP, DBP, TC, FMD | Yes |
| Lee 2008/China (Lee et al., 2008) | P/12 wk | Cranberry, extract, 1500 mg/day | NA | Placebo | 14/16, T2D patients | BMI, SBP, DBP, TC, TG, HDL-C, LDL-C, CRP | No |
| Li 2015/China (Li et al., 2015) | P/24 wk | Anthocyanin capsules | 320 | Placebo | 24/34, T2D patients | BMI, SBP, DBP, TC, TG, HDL-C, LDL-C, TNF-α | No |
| McAnulty 2005/US (McAnulty et al., 2005) | P/3 wk | Blueberry, fresh fruit, 250 g/day | NA | Others | 20, regular smokers | SBP, DBP | No |
| McAnulty 2014/US (McAnulty et al., 2014) | P/6 wk | Blueberry, freeze-dried powder, 38 g/day | NA | Placebo | 25, general population | BMI, SBP, DBP | No |
| Novotny 2015/US (Novotny et al., 2015) | P/8 wk | Cranberry, beverage, 480 mL/day | 20.6 | Placebo | 31/29, healthy | BMI, SBP, DBP, TC, TG, HDL-C, LDL-C, CRP | Yes |
| Nyberg 2013/Sweden (Nyberg et al., 2013) | C/4 wk | Blueberry, fruit, 150 g/day | NA | Others | 15/17, healthy | TG, HDL-C, LDL-C | No |
| Ohguro 2012/Japan (Ohguro et al., 2012) | P/24 mon | Anthocyanin capsules | 50 | Placebo | F/M: 17/23, with open-angle glaucoma | SBP, DBP | No |
| Qin 2009/China (Qin et al., 2009) | P/12 wk | Anthocyanin capsules | 320 | Placebo | 78/42, dyslipidemia | BMI, SBP, DBP, TC, TG, HDL-C, LDL-C | No |
| Riso 2013/Italy (Riso et al., 2013) | C/6 wk | Blueberry, freeze-dried powder, 25 g/day | 375 | Placebo | M:18, at elevated risk of CVD | BMI, SBP, DBP, TC, TG, HDL-C, LDL-C, CRP, TNF-α | Yes |
| Rodriguez-Mateos 2019/UK (Rodriguez-Mateos et al., 2019) | P/4 wk | Blueberry, freeze-dried powder, 11 g/day | 300 | Placebo | M: 40, healthy | FMD | Yes |
| Simão 2013/Brazil (Simão et al., 2013) | P/60 day | Cranberry, beverage, 700 mL/day | 28 | Others | 40/14, MetS patients | CRP, TNF-α | No |
| Skarpańska-Stejnborn 2017/Poland (Skarpańska-Stejnborn et al., 2017) | P/6 wk | Cranberry, extract, 720 mg/day | NA | Placebo | M:16, rowers | TNF-α | No |
| Stote 2020/US (Stote et al., 2020) | P/8 wk | Blueberry, freeze-dried powder, 22 g/day | 261.8 | Placebo | M:52, T2D patients | BMI, SBP, DBP, TC, TG, HDL-C, LDL-C, CRP | Yes |
| Stull 2010/US (Stull et al., 2010) | P/6 wk | Blueberry, freeze-dried powder, 45 g/day | 668 | Placebo | 27/5, with obese and insulin resistance | BMI, SBP, DBP, TC, TG, HDL-C, LDL-C, CRP, TNF-α | Yes |
| Stull 2015/US (Stull et al., 2015) | P/6 wk | Blueberry, freeze-dried powder, 45 g/day | 580.6 | Placebo | 28/16, MetS patients | BMI, SBP, DBP, TC, TG | Yes |
| Thompson 2017Ⅰ/Australia (Thompson et al., 2017b) | C/4 wk | Anthocyanin capsules | 320 | Placebo | 17/9, sedentary obese and overweight | BMI, SBP, DBP, TC, TG, CRP | No |
| Thompson 2017Ⅱ/Australia (Thompson et al., 2017a) | C/4 wk | Anthocyanin capsules | 320 | Placebo | 13/3, sedentary non-obese | BMI, SBP, DBP, TC, TG, CRP | No |
| Vidlar 2010/Czech (Vidlar et al., 2010) | P/6 mon | Cranberry, extract, 1500 mg/day | 1.65 | Others | M:42, at risk of prostate disease | TC, TG, CRP | No |
| Xu 2020/China (Xu et al., 2020) | P/12 wk | Anthocyanin capsules | 40/80/320 | Placebo | 130/46, dyslipidemia | BMI, SBP, DBP, TC, TG, HDL-C, LDL-C | No |
| Yang 2017/China (Yang et al., 2017) | P/12 wk | Anthocyanin capsules | 320 | Placebo | 106/54, with prediabetes and early untreated diabetes | BMI, SBP, DBP, TC, TG, HDL-C, LDL-C, CRP | No |
| Zare 2018/Iran (Zare Javid et al., 2018) | P/8 wk | Cranberry, beverage, 400 mL/day | 16 | Others | 14/7, T2D patients with periodontal disease | BMI, TC, TG, HDL-C, LDL-C | Yes |
| Zhang 2015/China (Zhang et al., 2015) | P/12 wk | Anthocyanin capsules | 320 | Placebo | 35/39, NAFLD patients | TC, TG, HDL-C, LDL-C | No |
| Zhang 2020/China (Zhang et al., 2020) | P/12 wk | Anthocyanin capsules | 40/80/320 | Placebo | 130/46, dyslipidemia | TC, CRP | No |
| Zhu 2011/China (Zhu et al., 2011) | P/12 wk | Anthocyanin capsules | 320 | Placebo | 87/63, hypercholesterolemia | BMI, SBP, DBP, TG, TC, HDL-C, LDL-CFMD | No |
| Zhu 2013/China (Zhu et al., 2013) | P/24 wk | Anthocyanin capsules | 320 | Placebo | 85/61, hypercholesterolemia | BMI, SBP, DBP, TC, TG, HDL-C, LDL-C, TNF-α | No |

^1^Abbreviations: C, crossover; CRP, C-reactive protein; CVD, cardiovascular disease; DBP, diastolic blood pressure; FMD, flow-mediated dilation; HDL-C, HDL cholesterol; LDL-C, LDL cholesterol; MetS, metabolic syndrome; mon, month; NA, not available; NAFLD, non-alcoholic fatty liver disease; P, parallel; SBP, systolic blood pressure; T2D, type 2 diabetes; TC, total cholesterol; TG, triglyceride; TNF-α, tumor necrosis factor alpha; wk, week.

1. **Table S3** Characteristics of included prospective cohort studies.^1^

| Study/country | Number of cases | Sample size (F/M) | Age, year | Follow-up, year | Diet assessment method | Covariate adjustments |
| --- | --- | --- | --- | --- | --- | --- |
| Adriouch 2018/French (Adriouch et al., 2018) | CHD incidence: 309  Stroke incidence:293  CVD incidence: 602 | 84158 (66227/17931) | 44.1 ±14.5 | 4.9 | 24 h dietary records | age, BMI, physical activity, smoking status, numbers of dietary records, alcohol intake, energy intake, family history of CVDs, educational level, season of completion of 24-h dietary records |
| Bondonno 2019/Australia (Bondonno et al., 2019) | CVD mortality: 3981 | 56048 (26666/29382) | 52-60 | 23 | FFQ | age, gender, BMI, smoking status, physical activity, alcohol intake, hypertension, hypercholesterolemia, social economic status, diabetes, prevalent disease, intakes of fish, red meat, processed meat, dietary fiber, polyunsaturated FAs, monounsaturated FAs, saturated FAs |
| Bondonno 2020/Australia (Bondonno et al., 2020) | CVD mortality: 213 | 2349 (971/1378) | 64.7 ± 9.2 | 14 | FFQ | age, gender, BMI, smoking status, physical activity, alcohol intake, hypertension, hypercholesterolemia, and social economic status |
| Cassidy 2012/US (Cassidy et al., 2012) | Stroke incidence: 1803 | F: 69622 | 30-55 | 14 | FFQ | age, BMI, physical activity, alcohol consumption, energy intake, use of multi-vitamin supplements, use of aspirin, menopausal status, smoking and history of type 2 diabetes, CHD, hypercholesterolemia, or hypertension |
| Cassidy 2013/US (Cassidy et al., 2013) | CHD incidence: 405 | F: 93600 | 25-42 | 18 | FFQ | age, BMI, physical activity, alcohol consumption, energy intake, cereal fibrin intake, fat intake, caffeine intake, use of aspirin, menopausal status, postmenopausal hormone use, oral contraceptive use, smoking, and family history of MI |
| Cassidy 2016/US (Cassidy et al., 2016) | Stroke incidence: 1572  CHD incidence: 4046 | M: 43880 | 39-77 | 24 | FFQ | lifestyle, diet, and health status, age, BMI, smoking, and family history |
| Goetz 2016/US (Goetz et al., 2016) | CHD incidence: 589 | 16678 | > 45 | 6.06 | FFQ | age, energy intake, gender, physical activity, smoking, race, region of residence, household income and educational attainment, energy intake from sweetened foods and beverages, reported beer, liquor and fat intake. |
| Ivey 2013/Australia (Ivey et al., 2013) | CVD mortality: 64 | F: 1063 | > 75 | 5 | FFQ | age, energy intake, BMI, previous atherosclerotic vascular disease, physical activity, previous diabetes, anti-hypertensive medication use, history of smoking and intakes of saturated fat, fiber, protein, starch, vitamin C and alcohol |
| Jacques 2015/US (Jacques et al., 2015) | CHD incidence: 261  CVD incidence: 518 | 2880 | 28-62 | 20 | FFQ | age, gender, smoking status, BMI, total energy intake and fruit/vegetable intake. |
| McCullough 2012/US (McCullough et al., 2012) | CVD mortality: 2771 | 98469 (60289/38180) | 69.5 | 7 | FFQ | age, smoking, beer and liquor intake, history of hypertension, history of cholesterol, family history of myocardial infarction, BMI, physical activity, energy intake, aspirin use, hormone replacement therapy, and gender. |
| Mink 2007/US (Mink et al., 2007) | Stroke mortality: 469  CHD mortality: 1329  CVD mortality: 2316 | F: 34489 | 55-69 | 16 | FFQ | age, energy intake, marital status, education, blood pressure, diabetes, BMI, waist-to-hip ratio, physical activity, smoking, and estrogen use |
| Mursu 2008/Finland (Mursu et al., 2008) | Stroke incidence: 153  CVD mortality: 153 | M: 1950 | 42-60 | 15.2 | 4-day food dietary record | age, examination years, BMI, systolic blood pressure, hypertension medication, serum HDL- and LDL-cholesterol, serum TG, maximal oxygen uptake, smoking, CVD in family, diabetes, alcohol intake, energy-adjusted intake of folate and vitamin E, total fat and saturated fat intake |
| Ponzo 2015/Italy (Ponzo et al., 2015) | CVD incidence: 125  CVD mortality: 84 | 1658 | 45-65 | 12 | FFQ | age, gender, BMI, education, living in a rural area, physical activity, fiber, and saturated fatty acid intakes, alcohol intake, smoking, values of systolic and diastolic blood pressure, total and HDL cholesterol, fasting glucose, CRP, statin, and aspirin use |
| Tresserra-Rimbau 2014/Spain (Tresserra-Rimbau et al., 2014) | CVD incidence: 273 | 7172 | 66 | 4.3 | FFQ | age, gender, smoking, BMI, alcohol, energy, physical activity, family history of CVD, aspirin use, antihypertensive drugs, cardiovascular drugs, and diabetes status, intake of proteins, saturated FAs, polyunsaturated FAs, monounsaturated FAs, and cholesterol. |
| Zamora-Ros 2013/Spain (Zamora-Ros et al., 2013) | CVD mortality: 416 | 40622 | 29-70 | 13.6 | 24 h dietary records | age, gender, BMI, education level, physical activity, smoking, alcohol intake, energy intake, vitamin C and fiber intakes. |

^1^Abbreviations: CHD, coronary heart disease; CRP, C-reactive protein; CVD, cardiovascular disease; FA, fatty acid; FFQ, food frequency questionnaire; MI, myocardial infarction; TG, triglyceride.

1. **Table S4** Summary of quality assessment for included randomized controlled trials.^1, 2^

|  | Item | Yes | No | NR |
| --- | --- | --- | --- | --- |
| Q1 | Was the study described as randomized, a randomized trial, a randomized clinical trial, or an RCT? | 44 | 0 | 0 |
| Q2 | Was the method of randomization adequate (i.e., use of randomly generated assignment)? | 19 | 1 | 24 |
| Q3 | Was the treatment allocation concealed (so that assignments could not be predicted)? | 35 | 1 | 8 |
| Q4 | Were study participants and providers blinded to treatment group assignment? | 33 | 1 | 10 |
| Q5 | Were the people assessing the outcomes blinded to the participants' group assignments? | 9 | 1 | 34 |
| Q6 | Were the groups similar at baseline on important characteristics that could affect outcomes (e.g., demographics, risk factors, co-morbid conditions)? | 34 | 1 | 9 |
| Q7 | Was the overall drop-out rate from the study at endpoint 20% or lower of the number allocated to treatment? | 41 | 3 | 0 |
| Q8 | Was the differential drop-out rate (between treatment groups) at endpoint 15 percentage points or lower? | 43 | 1 | 0 |
| Q9 | Was there high adherence to the intervention protocols for each treatment group? | 33 | 0 | 11 |
| Q10 | Were other interventions avoided or similar in the groups (e.g., similar background treatments)? | 44 | 0 | 0 |
| Q11 | Were outcomes assessed using valid and reliable measures, implemented consistently across all study participants? | 44 | 0 | 0 |
| Q12 | Did the authors report that the sample size was sufficiently large to be able to detect a difference in the main outcome between groups with at least 80% power? | 28 | 8 | 8 |
| Q13 | Were outcomes reported or subgroups analyzed prespecified (i.e., identified before analyses were conducted)? | 30 | 12 | 2 |
| Q14 | Were all randomized participants analyzed in the group to which they were originally assigned, i.e., did they use an intention-to-treat analysis? | 22 | 16 | 6 |

^1^Based on the National Heart, Lung, and Blood Institute Study Quality Assessment Tools of Controlled Intervention Studies.

^2^Abbreviation: NR, not reported.

1. **Table S5** Detailed results of quality assessment of included randomized controlled trials.^1, 2^

| Study | Q1 | Q2 | Q3 | Q4 | Q5 | Q6 | Q7 | Q8 | Q9 | Q10 | Q11 | Q12 | Q13 | Q14 | Score |
| --- | --- | --- | --- | --- | --- | --- | --- | --- | --- | --- | --- | --- | --- | --- | --- |
| Alnajjar 2019 (Alnajjar et al., 2019) | Y | NR | Y | Y | NR | NR | Y | Y | Y | Y | Y | Y | Y | Y | 11 |
| Arevström 2019 (Arevström et al., 2019) | Y | Y | Y | Y | NR | Y | Y | Y | Y | Y | Y | Y | Y | NR | 12 |
| Basu 2010 (Basu et al., 2010) | Y | NR | Y | Y | Y | Y | Y | Y | Y | Y | Y | Y | N | N | 11 |
| Basu 2011 (Basu et al., 2011) | Y | NR | NR | NR | Y | Y | N | Y | Y | Y | Y | Y | N | N | 8 |
| Chew 2019 (Chew et al., 2019) | Y | Y | Y | Y | NR | Y | Y | Y | Y | Y | Y | N | Y | Y | 12 |
| Curtis 2009 (Curtis et al., 2009) | Y | NR | Y | Y | NR | Y | Y | Y | Y | Y | Y | Y | N | Y | 11 |
| Curtis 2019 (Curtis et al., 2019) | Y | Y | Y | Y | NR | NR | Y | Y | Y | Y | Y | Y | Y | NR | 11 |
| Dohadwala 2011 (Dohadwala et al., 2011) | Y | NR | Y | Y | NR | Y | Y | Y | Y | Y | Y | Y | Y | N | 11 |
| Du 2019 (Du et al., 2019) | Y | NR | Y | Y | NR | Y | N | Y | Y | Y | Y | NR | Y | Y | 10 |
| Duthie 2006 (Duthie et al., 2006) | Y | NR | NR | NR | NR | NR | Y | Y | NR | Y | Y | N | N | Y | 6 |
| Flammer 2013 (Flammer et al., 2013) | Y | NR | Y | Y | Y | Y | Y | Y | Y | Y | Y | Y | Y | N | 12 |
| Guo 2020 (Guo et al., 2020) | Y | Y | Y | Y | Y | Y | Y | Y | Y | Y | Y | Y | Y | Y | 14 |
| Hassellund 2012 (Hassellund et al., 2012) | Y | Y | Y | Y | Y | N | Y | Y | Y | Y | Y | Y | Y | N | 12 |
| Hsia 2020 (Hsia et al., 2020) | Y | Y | Y | Y | NR | Y | Y | Y | Y | Y | Y | Y | Y | NR | 12 |
| Johnson 2015 (Johnson et al., 2015) | Y | Y | Y | Y | NR | Y | Y | Y | Y | Y | Y | Y | Y | Y | 13 |
| Johnson 2017 (Johnson et al., 2017) | Y | Y | Y | Y | NR | Y | Y | Y | Y | Y | Y | Y | Y | Y | 13 |
| Karlsen 2007 (Karlsen et al., 2007) | Y | NR | NR | NR | NR | Y | Y | Y | Y | Y | Y | N | N | N | 7 |
| Karlsen 2010 (Karlsen et al., 2010) | Y | NR | NR | NR | NR | Y | Y | Y | Y | Y | Y | N | N | N | 7 |
| Khan 2014 (Khan et al., 2014) | Y | Y | Y | Y | NR | NR | Y | Y | Y | Y | Y | Y | Y | N | 11 |
| Lee 2008 (Lee et al., 2008) | Y | NR | Y | Y | NR | Y | Y | Y | Y | Y | Y | N | N | Y | 10 |
| Li 2015 (Li et al., 2015) | Y | NR | Y | Y | NR | Y | Y | Y | NR | Y | Y | NR | Y | Y | 10 |
| McAnulty 2005 (McAnulty et al., 2005) | Y | NR | NR | NR | NR | Y | Y | Y | NR | Y | Y | Y | N | Y | 8 |
| McAnulty 2014 (McAnulty et al., 2014) | Y | NR | NR | NR | NR | Y | Y | Y | NR | Y | Y | NR | Y | Y | 8 |
| Novotny 2015 (Novotny et al., 2015) | Y | NR | Y | Y | NR | Y | Y | Y | NR | Y | Y | Y | Y | Y | 11 |
| Nyberg 2013 (Nyberg et al., 2013) | Y | NR | NR | NR | NR | Y | Y | Y | NR | Y | Y | NR | Y | N | 7 |
| Ohguro 2012 (Ohguro et al., 2012) | Y | Y | Y | Y | NR | Y | Y | Y | NR | Y | Y | Y | NR | N | 10 |
| Qin 2009 (Qin et al., 2009) | Y | NR | Y | Y | NR | Y | Y | Y | Y | Y | Y | N | N | Y | 10 |
| Riso 2013 (Riso et al., 2013) | Y | Y | Y | NR | NR | NR | Y | Y | NR | Y | Y | Y | Y | N | 9 |
| Rodriguez-Mateos 2019 (Rodriguez-Mateos et al., 2019) | Y | NR | Y | Y | NR | NR | Y | Y | NR | Y | Y | Y | Y | NR | 9 |
| Simão 2013 (Simão et al., 2013) | Y | N | N | N | NR | Y | Y | Y | Y | Y | Y | NR | N | N | 8 |
| Skarpańska-Stejnborn 2017 (Skarpańska-Stejnborn et al., 2017) | Y | Y | Y | NR | NR | Y | Y | Y | NR | Y | Y | NR | Y | Y | 10 |
| Stote 2020 (Stote et al., 2020) | Y | Y | Y | Y | Y | Y | Y | Y | Y | Y | Y | Y | Y | NR | 13 |
| Stull 2010 (Stull et al., 2010) | Y | NR | Y | Y | NR | Y | Y | Y | Y | Y | Y | N | N | Y | 10 |
| Stull 2015 (Stull et al., 2015) | Y | Y | Y | Y | NR | Y | Y | Y | Y | Y | Y | Y | Y | N | 12 |
| Thompson 2017Ⅰ (Thompson et al., 2017b) | Y | NR | Y | Y | NR | NR | Y | Y | Y | Y | Y | Y | Y | Y | 11 |
| Thompson 2017Ⅱ (Thompson et al., 2017a) | Y | NR | Y | Y | NR | NR | Y | Y | Y | Y | Y | Y | Y | Y | 11 |
| Vidlar 2010 (Vidlar et al., 2010) | Y | NR | Y | Y | NR | Y | Y | Y | NR | Y | Y | N | N | Y | 9 |
| Xu 2020 (Xu et al., 2020) | Y | Y | Y | Y | Y | Y | Y | Y | Y | Y | Y | Y | Y | Y | 14 |
| Yang 2017 (Yang et al., 2017) | Y | Y | Y | Y | Y | Y | Y | Y | Y | Y | Y | Y | Y | Y | 14 |
| Zare 2018 (Zare Javid et al., 2018) | Y | NR | NR | NR | NR | NR | N | N | Y | Y | Y | Y | Y | N | 6 |
| Zhang 2015 (Zhang et al., 2015) | Y | Y | Y | Y | Y | Y | Y | Y | Y | Y | Y | Y | Y | Y | 14 |
| Zhang 2020 (Zhang et al., 2020) | Y | Y | Y | Y | NR | Y | Y | Y | Y | Y | Y | NR | Y | NR | 11 |
| Zhu 2011 (Zhu et al., 2011) | Y | NR | Y | Y | NR | Y | Y | Y | Y | Y | Y | NR | NR | N | 9 |
| Zhu 2013 (Zhu et al., 2013) | Y | Y | Y | Y | N | Y | Y | Y | Y | Y | Y | Y | Y | N | 12 |

^1^Abbreviations: Y, yes; N, no; NR, not reported.

^2^Each question was related to the corresponding item in **Table S4**.

1. **Table S6** Summary of quality assessment for included prospective cohort studies.^1^

|  | Item | Yes | No | NR |
| --- | --- | --- | --- | --- |
| Q1 | Was the research question or objective in this paper clearly stated? | 15 | 0 | 0 |
| Q2 | Was the study population clearly specified and defined? | 13 | 2 | 0 |
| Q3 | Was the participation rate of eligible persons at least 50%? | 13 | 1 | 1 |
| Q4 | Were all the subjects selected or recruited from the same or similar populations (including the same time period)? Were inclusion and exclusion criteria for being in the study prespecified and applied uniformly to all participants? | 14 | 1 | 0 |
| Q5 | Was a sample size justification, power description, or variance and effect estimates provided? | 2 | 1 | 12 |
| Q6 | For the analyses in this paper, were the exposure(s) of interest measured prior to the outcome(s) being measured? | 15 | 0 | 0 |
| Q7 | Was the timeframe sufficient so that one could reasonably expect to see an association between exposure and outcome if it existed? | 15 | 0 | 0 |
| Q8 | For exposures that can vary in amount or level, did the study examine different levels of the exposure as related to the outcome (e.g., categories of exposure, or exposure measured as continuous variable)? | 12 | 3 | 0 |
| Q9 | Were the exposure measures (independent variables) clearly defined, valid, reliable, and implemented consistently across all study participants? | 15 | 0 | 0 |
| Q10 | Was the exposure(s) assessed more than once over time? | 14 | 0 | 1 |
| Q11 | Were the outcome measures (dependent variables) clearly defined, valid, reliable, and implemented consistently across all study participants? | 13 | 1 | 1 |
| Q12 | Were the outcome assessors blinded to the exposure status of participants? | 4 | 1 | 10 |
| Q13 | Was loss to follow-up after baseline 20% or less? | 10 | 2 | 3 |
| Q14 | Were key potential confounding variables measured and adjusted statistically for their impact on the relationship between exposure(s) and outcome(s)? | 15 | 0 | 0 |

^1^Based on the National Heart, Lung, and Blood Institute Study Quality Assessment Tools of Observational Cohort and Cross-Sectional Studies.

^2^Abbreviation: NR, not reported.

1. **Table S7** Detailed results of quality assessment of included prospective cohort studies.^1, 2^

| Study | Q1 | Q2 | Q3 | Q4 | Q5 | Q6 | Q7 | Q8 | Q9 | Q10 | Q11 | Q12 | Q13 | Q14 | Score |
| --- | --- | --- | --- | --- | --- | --- | --- | --- | --- | --- | --- | --- | --- | --- | --- |
| Adriouch 2018 (Adriouch et al., 2018) | Y | N | Y | Y | Y | Y | Y | Y | Y | Y | Y | NR | Y | Y | 12 |
| Bondonno 2019 (Bondonno et al., 2019) | Y | Y | Y | Y | Y | Y | Y | Y | Y | Y | Y | NR | Y | Y | 13 |
| Bondonno 2020 (Bondonno et al., 2020) | Y | Y | Y | Y | NR | Y | Y | Y | Y | Y | Y | NR | N | Y | 11 |
| Cassidy 2012 (Cassidy et al., 2012) | Y | Y | Y | Y | NR | Y | Y | Y | Y | Y | Y | Y | Y | Y | 13 |
| Cassidy 2013 (Cassidy et al., 2013) | Y | Y | Y | Y | NR | Y | Y | Y | Y | Y | Y | NR | Y | Y | 12 |
| Cassidy 2016 (Cassidy et al., 2016) | Y | N | Y | Y | NR | Y | Y | Y | Y | Y | Y | Y | Y | Y | 12 |
| Goetz 2016 (Goetz et al., 2016) | Y | Y | Y | Y | N | Y | Y | Y | Y | Y | Y | N | N | Y | 11 |
| Ivey 2013 (Ivey et al., 2013) | Y | Y | NR | Y | NR | Y | Y | Y | Y | NR | NR | NR | Y | Y | 9 |
| Jacques 2015 (Jacques et al., 2015) | Y | Y | Y | N | NR | Y | Y | N | Y | Y | Y | NR | Y | Y | 10 |
| McCullough 2012 (McCullough et al., 2012) | Y | Y | Y | Y | NR | Y | Y | Y | Y | Y | Y | NR | Y | Y | 12 |
| Mink 2007 (Mink et al., 2007) | Y | Y | N | Y | NR | Y | Y | Y | Y | Y | Y | NR | NR | Y | 10 |
| Mursu 2008 (Mursu et al., 2008) | Y | Y | Y | Y | NR | Y | Y | N | Y | Y | Y | NR | Y | Y | 11 |
| Ponzo 2015 (Ponzo et al., 2015) | Y | Y | Y | Y | NR | Y | Y | N | Y | Y | Y | Y | NR | Y | 11 |
| Tresserra-Rimbau 2014 (Tresserra-Rimbau et al., 2014) | Y | Y | Y | Y | NR | Y | Y | Y | Y | Y | N | Y | NR | Y | 11 |
| Zamora-Ros 2013 (Zamora-Ros et al., 2013) | Y | Y | Y | Y | NR | Y | Y | Y | Y | Y | Y | NR | Y | Y | 12 |

^1^Abbreviations: Y, yes; N, no; NR, not reported.

^2^Each question was related to the corresponding item in **Table S6**.

1. **Table S8** Pooled effects of purified anthocyanins and anthocyanin-rich berries on BMI.^1, 2^

| Variables | Number of comparisons (subjects) | WMD (95% CI), kg/m^2^ | *P*_difference_ | *I*^2^, % | *P*_heterogeneity_ |
| --- | --- | --- | --- | --- | --- |
| *Purified anthocyanins* | | | | | |
| Overall | 15 (901) | 0.07 (-0.09, 0.23) | 0.375 | 0.0 | 0.995 |
| Study design | | | | | |
| Parallel | 13 (819) | 0.08 (-0.09, 0.24) | 0.358 | 0.0 | 0.985 |
| Crossover | 2 (82) | -0.07 (-1.09, 0.95) | 0.894 | 0.0 | 0.890 |
| Duration | | | | | |
| < 8 weeks | 7 (189) | -0.14 (-0.60, 0.32) | 0.558 | 0.0 | 1.000 |
| ≥ 8 weeks | 8 (712) | 0.10 (-0.07, 0.27) | 0.247 | 0.0 | 0.886 |
| Health status of subjects | | | | | |
| Low CVD risk | 6 (139) | -0.15 (-0.61, 0.32) | 0.543 | 0.0 | 1.000 |
| High CVD risk | 9 (762) | 0.10 (-0.07, 0.27) | 0.246 | 0.0 | 0.935 |
| Type of control | | | | | |
| Placebo | 15 (901) | 0.07 (-0.09, 0.23) | 0.375 | 0.0 | 0.995 |
| Dose of anthocyanins | | | | | |
| < 200 mg/day | 6 (221) | 0.08 (-0.13, 0.29) | 0.439 | 0.0 | 0.868 |
| ≥ 200 mg/day | 9 (680) | 0.06 (-0.19, 0.30) | 0.647 | 0.0 | 0.977 |
| Study quality | | | | | |
| Low to moderate | 2 (178) | -0.10 (-1.34, 1.14) | 0.871 | 0.0 | 0.980 |
| High | 13 (723) | 0.08 (-0.09, 0.23) | 0.360 | 0.0 | 0.984 |
| Received industry funding? | | | | | |
| Yes | 1 (52) | -0.10 (-2.06, 1.86) | 0.920 | - | - |
| No | 14 (849) | 0.07 (-0.09, 0.23) | 0.369 | 0.0 | 0.991 |
| *Anthocyanin-rich berries* | | | | | |
| Overall | 13 (498) | 0.06 (-0.03, 0.15) | 0.202 | 0.0 | 0.807 |
| Type of berry | | | | | |
| Blueberry | 7 (273) | 0.09 (-0.18, 0.36) | 0.522 | 0.0 | 0.996 |
| Cranberry | 3 (111) | -0.30 (-0.57, -0.02) | 0.035 | 0.0 | 0.994 |
| Blackcurrant | 2 (64) | -0.02 (-2.67, 2.64) | 0.991 | 0.0 | 0.942 |
| Bilberry | 1 (50) | 0.10 (0.00, 0.20) | 0.050 | - | - |
| Study design | | | | | |
| Parallel | 12 (462) | 0.06 (-0.03, 0.15) | 0.199 | 0.0 | 0.741 |
| Crossover | 1 (36) | -0.10 (-1.80, 1.60) | 0.908 | - | - |
| Duration | | | | | |
| < 8 weeks | 6 (201) | 0.09 (-0.18, 0.36) | 0.516 | 0.0 | 1.000 |
| ≥ 8 weeks | 7 (297) | -0.04 (-0.26, 0.18) | 0.727 | 20.2 | 0.276 |
| Health status of subjects | | | | | |
| Low CVD risk | 5 (193) | -0.30 (-1.70, 1.10) | 0.671 | 0.0 | 0.994 |
| High CVD risk | 8 (305) | 0.05 (-0.06, 0.15) | 0.384 | 3.2 | 0.405 |
| Type of control | | | | | |
| Placebo | 11 (427) | -0.10 (-0.29, 0.09) | 0.313 | 0.0 | 0.924 |
| Others | 2 (71) | 0.10 (0.00, 0.20) | 0.050 | 0.0 | 0.917 |
| Study quality | | | | | |
| Low to moderate | 6 (188) | -0.30 (-0.57, -0.02) | 0.033 | 0.0 | 0.998 |
| High | 7 (310) | 0.10 (0.01, 0.19) | 0.037 | 0.0 | 1.000 |
| Received industry funding? | | | | | |
| Yes | 10 (393) | 0.08 (-0.18, 0.35) | 0.533 | 0.0 | 1.000 |
| No | 3 (105) | -0.08 (-0.44, 0.28) | 0.672 | 71.7 | 0.029 |
| Type of intervention | | | | | |
| Powder | 9 (353) | 0.06 (-0.03, 0.15) | 0.198 | 0.0 | 0.469 |
| Beverage | 4 (142) | -0.13 (-1.71, 1.45) | 0.871 | 0.0 | 0.999 |

^1^Abbreviations: CVD, cardiovascular disease; WMD, weighted mean difference.

^2^The pooled effects sizes and 95% CIs were calculated using the random-effects model. Between-study heterogeneity was examined using the Cochrane’s Q test.

1. **Table S9** Pooled effects of purified anthocyanins and anthocyanin-rich berries on systolic blood pressure.^1, 2^

| Variables | Number of comparisons (subjects) | WMD (95% CI), mmHg | *P*_difference_ | *I*^2^, % | *P*_heterogeneity_ |
| --- | --- | --- | --- | --- | --- |
| *Purified anthocyanins* | | | | | |
| Overall | 17 (995) | -0.11 (-1.65, 1.44) | 0.893 | 0.0 | 0.832 |
| Study design | | | | | |
| Parallel | 14 (859) | 0.21 (-1.52, 1.94) | 0.812 | 0.0 | 0.840 |
| Crossover | 3 (136) | -1.34 (-4.75, 2.08) | 0.443 | 0.0 | 0.377 |
| Duration | | | | | |
| < 8 weeks | 8 (243) | 0.02 (-2.47, 2.50) | 0.989 | 0.0 | 0.802 |
| ≥ 8 weeks | 9 (752) | -0.18 (-2.15, 1.78) | 0.856 | 0.0 | 0.557 |
| Health status of subjects | | | | | |
| Low CVD risk | 7 (179) | 0.23 (-2.57, 3.02) | 0.874 | 0.0 | 0.713 |
| High CVD risk | 10 (816) | -0.25 (-2.10, 1.60) | 0.791 | 0.0 | 0.656 |
| Type of control | | | | | |
| Placebo | 17 (995) | -0.11 (-1.65, 1.44) | 0.893 | 0.0 | 0.832 |
| Dose of anthocyanins | | | | | |
| < 200 mg/day | 7 (261) | 0.41 (-2.01, 2.83) | 0.739 | 0.0 | 0.990 |
| ≥ 200 mg/day | 10 (734) | -0.41 (-2.47, 1.65) | 0.696 | 4.9 | 0.395 |
| Study quality | | | | | |
| Low to moderate | 3 (218) | 2.61 (-2.25, 7.46) | 0.293 | 0.0 | 0.875 |
| High | 14 (777) | -0.41 (-2.03, 1.22) | 0.622 | 0.0 | 0.771 |
| Received industry funding? | | | | | |
| Yes | 2 (106) | 4.04 (-0.93, 9.02) | 0.111 | 0.0 | 0.333 |
| No | 15 (889) | -0.55 (-2.17, 1.08) | 0.509 | 0.0 | 0.944 |
| *Anthocyanin-rich berries* | | | | | |
| Overall | 20 (883) | -0.64 (-1.82, 0.53) | 0.284 | 77.2 | <0.001 |
| Type of berry | | | | | |
| Blueberry | 11 (456) | 0.78 (-0.44, 2.00) | 0.210 | 42.3 | 0.068 |
| Cranberry | 6 (313) | -2.08 (-5.33, 1.18) | 0.210 | 58.2 | 0.035 |
| Blackcurrant | 2 (64) | 4.57 (-1.53, 10.68) | 0.142 | 0.0 | 0.631 |
| Bilberry | 1 (50) | -5.80 (-8.56, -3.05) | <0.001 | - | - |
| Study design | | | | | |
| Parallel | 18 (759) | -0.70 (-1.92, 0.51) | 0.255 | 79.6 | <0.001 |
| Crossover | 2 (124) | 0.32 (-5.62, 6.27) | 0.915 | 0.0 | 0.877 |
| Duration | | | | | |
| < 8 weeks | 8 (306) | 1.07 (-1.96, 4.09) | 0.490 | 0.0 | 0.878 |
| ≥ 8 weeks | 12 (577) | -1.00 (-2.31, 0.31) | 0.135 | 86.2 | <0.001 |
| Health status of subjects | | | | | |
| Low CVD risk | 5 (196) | -2.58 (-9.08, 3.93) | 0.437 | 68.4 | 0.013 |
| High CVD risk | 15 (687) | -0.29 (-1.46, 0.88) | 0.623 | 79.3 | <0.001 |
| Type of control | | | | | |
| Placebo | 17 (765) | 0.22 (-0.93, 1.37) | 0.702 | 74.8 | <0.001 |
| Others | 3 (118) | -5.68 (-8.19, -3.16) | <0.001 | 0.0 | 0.917 |
| Study quality | | | | | |
| Low to moderate | 7 (238) | 0.00 (-0.01, 0.01) | 0.998 | 0.0 | 0.470 |
| High | 13 (645) | -1.00 (-2.79, 0.80) | 0.277 | 76.8 | <0.001 |
| Received industry funding? | | | | | |
| Yes | 16 (758) | -0.04 (-1.51, 1.43) | 0.956 | 54.7 | 0.005 |
| No | 4 (125) | -2.72 (-6.99, 1.56) | 0.213 | 83.1 | <0.001 |
| Type of intervention | | | | | |
| Powder | 12 (516) | -0.40 (-1.66, 0.86) | 0.532 | 84.0 | <0.001 |
| Beverage | 7 (347) | -1.28 (-5.40, 2.84) | 0.543 | 54.6 | 0.040 |
| Fresh fruits | 1 (20) | -3.36 (-14.55, 7.83) | 0.556 | - | - |

^1^Abbreviations: CVD, cardiovascular disease; WMD, weighted mean difference.

^2^The pooled effects sizes and 95% CIs were calculated using the random-effects model. Between-study heterogeneity was examined using the Cochrane’s Q test.

1. **Table S10** Pooled effects of purified anthocyanins and anthocyanin-rich berries on diastolic blood pressure.^1, 2^

| Variables | Number of comparisons (subjects) | WMD (95% CI), mmHg | *P*_difference_ | *I*^2^, % | *P*_heterogeneity_ |
| --- | --- | --- | --- | --- | --- |
| *Purified anthocyanins* | | | | | |
| Overall | 17 (995) | 0.74 (-0.25, 1.72) | 0.143 | 0.0 | 0.979 |
| Study design | | | | | |
| Parallel | 14 (859) | 0.74 (-0.34, 1.82) | 0.178 | 0.0 | 0.935 |
| Crossover | 3 (136) | 0.70 (-1.68, 3.08) | 0.564 | 0.0 | 0.841 |
| Duration | | | | | |
| < 8 weeks | 8 (243) | 1.88 (0.25, 3.51) | 0.023 | 0.0 | 0.880 |
| ≥ 8 weeks | 9 (752) | 0.08 (-1.16, 1.31) | 0.905 | 0.0 | 1.000 |
| Health status of subjects | | | | | |
| Low CVD risk | 7 (179) | 2.10 (0.17, 4.04) | 0.033 | 0.0 | 0.798 |
| High CVD risk | 10 (816) | 0.26 (-0.88, 1.40) | 0.655 | 0.0 | 0.999 |
| Type of control | | | | | |
| Placebo | 17 (995) | 0.74 (-0.25, 1.72) | 0.143 | 0.0 | 0.979 |
| Dose of anthocyanins | | | | | |
| < 200 mg/day | 7 (261) | 1.23 (-0.32, 2.77) | 0.121 | 0.0 | 0.684 |
| ≥ 200 mg/day | 10 (734) | 0.40 (-0.87, 1.68) | 0.536 | 0.0 | 0.991 |
| Study quality | | | | | |
| Low to moderate | 3 (218) | 0.46 (-2.56, 3.47) | 0.766 | 0.0 | 0.932 |
| High | 14 (777) | 0.77 (-0.27, 1.81) | 0.148 | 0.0 | 0.927 |
| Received industry funding? | | | | | |
| Yes | 2 (106) | 1.00 (-1.54, 3.54) | 0.440 | 0.0 | 1.000 |
| No | 15 (889) | 0.69 (-0.38, 1.75) | 0.206 | 0.0 | 0.949 |
| *Anthocyanin-rich berries* | | | | | |
| Overall | 20 (885) | -0.96 (-2.13, 0.21) | 0.107 | 79.5 | <0.001 |
| Type of berry | | | | | |
| Blueberry | 11 (458) | -1.16 (-2.61, 0.29) | 0.116 | 83.4 | <0.001 |
| Cranberry | 6 (313) | 0.06 (-3.86, 3.97) | 0.976 | 78.8 | <0.001 |
| Blackcurrant | 2 (64) | 2.48 (-3.90, 8.86) | 0.446 | 0.0 | 0.645 |
| Bilberry | 1 (50) | -2.80 (-4.23, -1.37) | <0.001 | - | - |
| Study design | | | | | |
| Parallel | 18 (761) | -1.10 (-2.34, 0.14) | 0.083 | 81.5 | <0.001 |
| Crossover | 2 (124) | 0.46 (-2.57, 3.49) | 0.765 | 0.0 | 0.609 |
| Duration | | | | | |
| < 8 weeks | 8 (306) | 0.18 (-1.93, 2.29) | 0.867 | 0.0 | 0.815 |
| ≥ 8 weeks | 12 (579) | -1.25 (-2.62, 0.11) | 0.072 | 87.5 | <0.001 |
| Health status of subjects | | | | | |
| Low CVD risk | 5 (198) | -3.28 (-7.58, 1.02) | 0.135 | 52.1 | 0.080 |
| High CVD risk | 15 (687) | -0.47 (-1.68, 0.73) | 0.440 | 81.5 | <0.001 |
| Type of control | | | | | |
| Placebo | 17 (767) | -0.56 (-1.86, 0.74) | 0.397 | 81.0 | <0.001 |
| Others | 3 (118) | -2.84 (-4.19, -1.49) | <0.001 | 0.0 | 0.986 |
| Study quality | | | | | |
| Low to moderate | 7 (240) | -1.96 (-4.27, 0.35) | 0.096 | 0.0 | 0.858 |
| High | 13 (645) | -0.71 (-2.05, 0.63) | 0.297 | 86.6 | <0.001 |
| Received industry funding? | | | | | |
| Yes | 16 (760) | -0.67 (-1.97, 0.62) | 0.307 | 82.0 | <0.001 |
| No | 4 (125) | -2.69 (-4.07, -1.32) | <0.001 | 0.0 | 0.540 |
| Type of intervention | | | | | |
| Powder | 12 (518) | -1.33 (-2.64, -0.01) | 0.048 | 83.8 | <0.001 |
| Beverage | 7 (347) | 0.28 (-3.43, 3.99) | 0.882 | 75.0 | 0.001 |
| Fresh fruits | 1 (20) | -2.80 (-13.78, 8.18) | 0.617 | - | - |

^1^Abbreviations: CVD, cardiovascular disease; WMD, weighted mean difference.

^2^The pooled effects sizes and 95% CIs were calculated using the random-effects model. Between-study heterogeneity was examined using the Cochrane’s Q test.

1. **Table S11** Pooled effects of purified anthocyanins and anthocyanin-rich berries on flow-mediated dilation.^1, 2^

| Variables | Number of comparisons (subjects) | WMD (95% CI), % | *P*_difference_ | *I*^2^, % | *P*_heterogeneity_ |
| --- | --- | --- | --- | --- | --- |
| *Purified anthocyanins* | | | | | |
| Overall | 1 (146) | 2.66 (2.02, 3.30) | <0.001 | - | - |
| *Anthocyanin-rich berries* | | | | | |
| Overall | 6 (263) | 1.20 (-0.21, 2.60) | 0.096 | 98.7 | <0.001 |
| Type of berry | | | | | |
| Blueberry | 3 (111) | 1.10 (-0.83, 3.04) | 0.263 | 99.5 | <0.001 |
| Cranberry | 1 (88) | 0.30 (-1.45, 2.05) | 0.736 | - | - |
| Blackcurrant | 2 (64) | 1.78 (0.67, 2.90) | 0.002 | 0.0 | 0.707 |
| Study design | | | | | |
| Parallel | 5 (175) | 1.35 (-0.18, 2.87) | 0.083 | 99.0 | <0.001 |
| Crossover | 1 (88) | 0.30 (-1.45, 2.05) | 0.736 | - | - |
| Duration | | | | | |
| < 8 weeks | 4 (192) | 1.85 (0.78, 2.92) | 0.001 | 68.8 | 0.022 |
| ≥ 8 weeks | 2 (71) | 0.31 (-1.10, 1.72) | 0.667 | 97.6 | <0.001 |
| Health status of subjects | | | | | |
| Low CVD risk | 3 (104) | 2.49 (1.95, 3.04) | <0.001 | 22.3 | 0.276 |
| High CVD risk | 3 (159) | 0.31 (-0.90, 1.51) | 0.617 | 95.2 | <0.001 |
| Type of control | | | | | |
| Placebo | 6 (263) | 1.20 (-0.21, 2.60) | 0.096 | 98.7 | <0.001 |
| Study quality | | | | | |
| Low to moderate | 1 (40) | 2.68 (2.56, 2.80) | <0.001 | - | - |
| High | 5 (223) | 0.79 (-0.21, 1.78) | 0.122 | 91.6 | <0.001 |
| Received industry funding? | | | | | |
| Yes | 6 (263) | 1.20 (-0.21, 2.60) | 0.096 | 98.7 | <0.001 |
| Type of intervention | | | | | |
| Powder | 3 (111) | 1.10 (-0.83, 3.04) | 0.263 | 99.5 | <0.001 |
| Beverage | 3 (152) | 1.35 (0.39, 2.31) | 0.006 | 4.1 | 0.352 |

^1^Abbreviations: CVD, cardiovascular disease; WMD, weighted mean difference.

^2^The pooled effects sizes and 95% CIs were calculated using the random-effects model. Between-study heterogeneity was examined using the Cochrane’s Q test.

1. **Table S12** Pooled effects of purified anthocyanins and anthocyanin-rich berries on circulating HDL cholesterol.^1, 2^

| Variables | Number of comparisons (subjects) | WMD (95% CI), mg/dL | *P*_difference_ | *I*^2^, % | *P*_heterogeneity_ |
| --- | --- | --- | --- | --- | --- |
| *Purified anthocyanins* | | | | | |
| Overall | 14 (893) | 11.49 (7.43, 15.55) | <0.001 | 93.5 | <0.001 |
| Study design | | | | | |
| Parallel | 14 (893) | 11.49 (7.43, 15.55) | <0.001 | 93.5 | <0.001 |
| Duration | | | | | |
| < 8 weeks | 5 (107) | 30.88 (26.93, 34.83) | <0.001 | 0.0 | 0.994 |
| ≥ 8 weeks | 9 (786) | 2.76 (1.34, 4.18) | <0.001 | 43.8 | 0.076 |
| Health status of subjects | | | | | |
| Low CVD risk | 5 (107) | 30.88 (26.93, 34.83) | <0.001 | 0.0 | 0.994 |
| High CVD risk | 9 (786) | 2.76 (1.34, 4.18) | <0.001 | 43.8 | 0.076 |
| Type of control | | | | | |
| Placebo | 14 (893) | 11.49 (7.43, 15.55) | <0.001 | 93.5 | <0.001 |
| Dose of anthocyanins | | | | | |
| < 200 mg/day | 6 (221) | 20.66 (9.88, 31.44) | <0.001 | 96.7 | <0.001 |
| ≥ 200 mg/day | 8 (672) | 5.24 (1.87, 8.61) | 0.002 | 83.8 | <0.001 |
| Study quality | | | | | |
| Low to moderate | 2 (178) | 4.90 (1.75, 8.06) | 0.002 | 0.0 | 0.432 |
| High | 12 (715) | 12.49 (7.87, 17.11) | <0.001 | 94.5 | <0.001 |
| Received industry funding? | | | | | |
| Yes | 1 (52) | 0.00 (-7.43, 7.43) | 1.000 | - | - |
| No | 13 (841) | 12.34 (8.10, 16.58) | <0.001 | 93.9 | <0.001 |
| *Anthocyanin-rich berries* | | | | | |
| Overall | 14 (620) | 0.50 (-0.64, 1.65) | 0.389 | 77.7 | <0.001 |
| Type of berry | | | | | |
| Blueberry | 7 (309) | 1.46 (0.20, 2.72) | 0.023 | 85.9 | <0.001 |
| Cranberry | 5 (230) | -1.81 (-4.28, 0.66) | 0.151 | 0.0 | 0.722 |
| Bilberry | 2 (81) | -2.64 (-5.96, 0.68) | 0.119 | 0.0 | 0.379 |
| Study design | | | | | |
| Parallel | 10 (438) | 1.02 (-0.19, 2.23) | 0.097 | 81.4 | <0.001 |
| Crossover | 4 (182) | -2.09 (-4.57, 0.38) | 0.097 | 0.0 | 0.600 |
| Duration | | | | | |
| < 8 weeks | 5 (214) | -1.80 (-4.15, 0.54) | 0.132 | 0.0 | 0.664 |
| ≥ 8 weeks | 9 (406) | 1.02 (-0.21, 2.26) | 0.105 | 83.5 | <0.001 |
| Health status of subjects | | | | | |
| Low CVD risk | 2 (86) | 1.29 (-3.39, 5.97) | 0.589 | 0.0 | 0.658 |
| High CVD risk | 12 (534) | 0.44 (-0.76, 1.64) | 0.473 | 81.1 | <0.001 |
| Type of control | | | | | |
| Placebo | 10 (476) | 0.71 (-0.58, 2.00) | 0.280 | 83.1 | <0.001 |
| Others | 4 (144) | -0.23 (-2.17, 1.71) | 0.815 | 0.0 | 0.531 |
| Study quality | | | | | |
| Low to moderate | 6 (193) | -0.38 (-2.21, 1.45) | 0.681 | 0.0 | 0.679 |
| High | 8 (427) | 0.83 (-0.50, 2.15) | 0.219 | 86.3 | <0.001 |
| Received industry funding? | | | | | |
| Yes | 10 (483) | 0.92 (-0.28, 2.11) | 0.132 | 81.9 | <0.001 |
| No | 4 (137) | -2.37 (-5.30, 0.56) | 0.113 | 0.0 | 0.555 |
| Type of intervention | | | | | |
| Powder | 9 (394) | 0.87 (-0.39, 2.12) | 0.175 | 88.4 | <0.001 |
| Beverage | 4 (200) | -1.56 (-4.18, 1.05) | 0.241 | 0.0 | 0.624 |
| Fresh fruits | 1 (26) | 3.48 (-7.28, 14.24) | 0.526 | - | - |

^1^Abbreviations: CVD, cardiovascular disease; WMD, weighted mean difference.

^2^The pooled effects sizes and 95% CIs were calculated using the random-effects model. Between-study heterogeneity was examined using the Cochrane’s Q test.

1. **Table S13** Pooled effects of purified anthocyanins and anthocyanin-rich berries on circulating total cholesterol.^1, 2^

| Variables | Number of comparisons (subjects) | WMD (95% CI), mg/dL | *P*_difference_ | *I*^2^, % | *P*_heterogeneity_ |
| --- | --- | --- | --- | --- | --- |
| *Purified anthocyanins* | | | | | |
| Overall | 16 (975) | -2.17 (-5.74, 1.40) | 0.234 | 0.0 | 0.814 |
| Study design | | | | | |
| Parallel | 14 (893) | -1.87 (-5.52, 1.79) | 0.317 | 0.0 | 0.739 |
| Crossover | 2 (82) | -8.81 (-25.88, 8.26) | 0.312 | 0.0 | 0.853 |
| Duration | | | | | |
| < 8 weeks | 7 (189) | 2.07 (-4.95, 9.08) | 0.563 | 0.0 | 0.899 |
| ≥ 8 weeks | 9 (786) | -3.65 (-7.80, 0.50) | 0.085 | 0.0 | 0.650 |
| Health status of subjects | | | | | |
| Low CVD risk | 6 (139) | 3.36 (-4.10, 10.83) | 0.377 | 0.0 | 0.942 |
| High CVD risk | 10 (836) | -3.81 (-7.88, 0.26) | 0.066 | 0.0 | 0.728 |
| Type of control | | | | | |
| Placebo | 16 (975) | -2.17 (-5.74, 1.40) | 0.234 | 0.0 | 0.814 |
| Dose of anthocyanins | | | | | |
| < 200 mg/day | 6 (221) | 0.50 (-5.01, 6.02) | 0.858 | 0.0 | 0.891 |
| ≥ 200 mg/day | 10 (754) | -4.10 (-8.79, 0.59) | 0.087 | 0.0 | 0.653 |
| Study quality | | | | | |
| Low to moderate | 2 (178) | -3.84 (-16.78, 9.11) | 0.561 | 0.0 | 0.967 |
| High | 14 (797) | -2.03 (-5.75, 1.68) | 0.284 | 0.0 | 0.693 |
| Received industry funding? | | | | | |
| Yes | 1 (52) | 11.60 (-4.13, 27.33) | 0.148 | - | - |
| No | 15 (923) | -2.92 (-6.59, 0.75) | 0.119 | 0.0 | 0.935 |
| *Anthocyanin-rich berries* | | | | | |
| Overall | 20 (895) | -4.48 (-8.94, -0.02) | 0.049 | 86.4 | <0.001 |
| Type of berry | | | | | |
| Blueberry | 7 (327) | 0.43 (-5.00, 5.85) | 0.878 | 84.4 | <0.001 |
| Cranberry | 8 (361) | -4.45 (-13.81, 4.90) | 0.351 | 69.6 | 0.002 |
| Blackcurrant | 2 (64) | 1.91 (-12.03, 15.84) | 0.789 | 0.0 | 0.786 |
| Bilberry | 3 (143) | -11.99 (-25.11, 1.14) | 0.074 | 85.5 | 0.001 |
| Study design | | | | | |
| Parallel | 17 (739) | -4.59 (-9.46, 0.27) | 0.064 | 88.3 | <0.001 |
| Crossover | 3 (156) | -4.53 (-11.67, 2.62) | 0.214 | 0.0 | 0.901 |
| Duration | | | | | |
| < 8 weeks | 9 (378) | -5.04 (-9.82, -0.25) | 0.039 | 0.0 | 0.934 |
| ≥ 8 weeks | 11 (517) | -4.84 (-10.69, 1.02) | 0.105 | 92.3 | <0.001 |
| Health status of subjects | | | | | |
| Low CVD risk | 5 (186) | 3.56 (-5.19, 12.30) | 0.425 | 0.0 | 0.991 |
| High CVD risk | 15 (709) | -6.09 (-11.09, -1.08) | 0.017 | 89.9 | <0.001 |
| Type of control | | | | | |
| Placebo | 15 (673) | -2.75 (-6.95, 1.46) | 0.200 | 82.4 | <0.001 |
| Others | 5 (222) | -6.88 (-20.85, 7.10) | 0.335 | 70.9 | 0.008 |
| Study quality | | | | | |
| Low to moderate | 8 (291) | -5.71 (-16.37, 4.96) | 0.294 | 53.3 | 0.036 |
| High | 12 (604) | -3.50 (-8.42, 1.42) | 0.163 | 90.1 | <0.001 |
| Received industry funding? | | | | | |
| Yes | 15 (680) | 0.35 (-3.37, 4.06) | 0.856 | 70.5 | <0.001 |
| No | 5 (215) | -13.19 (-23.93, -2.45) | 0.016 | 81.1 | <0.001 |
| Type of intervention | | | | | |
| Powder | 11 (480) | -6.77 (-13.16, -0.39) | 0.038 | 92.5 | <0.001 |
| Beverage | 9 (415) | -1.38 (-4.16, 1.40) | 0.330 | 0.0 | 0.956 |

^1^Abbreviations: CVD, cardiovascular disease; WMD, weighted mean difference.

^2^The pooled effects sizes and 95% CIs were calculated using the random-effects model. Between-study heterogeneity was examined using the Cochrane’s Q test.

1. **Table S14** Pooled effects of purified anthocyanins and anthocyanin-rich berries on circulating tumor necrosis factor alpha.^1, 2^

| Variables | Number of comparisons (subjects) | WMD (95% CI), pg/mL | *P*_difference_ | *I*^2^, % | *P*_heterogeneity_ |
| --- | --- | --- | --- | --- | --- |
| *Purified anthocyanins* | | | | | |
| Overall | 9 (481) | -1.62 (-2.76, -0.48) | 0.005 | 0.0 | 0.952 |
| Study design | | | | | |
| Parallel | 9 (481) | -1.62 (-2.76, -0.48) | 0.005 | 0.0 | 0.952 |
| Duration | | | | | |
| < 8 weeks | 6 (225) | -2.05 (-3.57, -0.53) | 0.008 | 0.0 | 0.926 |
| ≥ 8 weeks | 3 (256) | -1.07 (-2.80, 0.66) | 0.225 | 0.0 | 0.733 |
| Health status of subjects | | | | | |
| Low CVD risk | 6 (225) | -2.05 (-3.57, -0.53) | 0.008 | 0.0 | 0.926 |
| High CVD risk | 3 (256) | -1.07 (-2.80, 0.66) | 0.225 | 0.0 | 0.733 |
| Type of control | | | | | |
| Placebo | 9 (481) | -1.62 (-2.76, -0.48) | 0.005 | 0.0 | 0.952 |
| Dose of anthocyanins | | | | | |
| < 200 mg/day | 4 (88) | -2.46 (-4.93, 0.01) | 0.051 | 0.0 | 0.951 |
| ≥ 200 mg/day | 5 (393) | -1.40 (-2.68, -0.11) | 0.033 | 0.0 | 0.772 |
| Study quality | | | | | |
| Low to moderate | 2 (176) | -2.11 (-4.11, -0.10) | 0.039 | 0.0 | 0.762 |
| High | 7 (305) | -1.39 (-2.78, -0.01) | 0.049 | 0.0 | 0.892 |
| Received industry funding? | | | | | |
| Yes | 1 (52) | -3.00 (-8.13, 2.13) | 0.251 | - | - |
| No | 8 (429) | -1.55 (-2.72, -0.38) | 0.009 | 0.0 | 0.934 |
| *Anthocyanin-rich berries* | | | | | |
| Overall | 10 (460) | 0.10 (-0.15, 0.35) | 0.436 | 0.0 | 0.606 |
| Type of berry | | | | | |
| Blueberry | 4 (147) | 0.04 (-0.30, 0.39) | 0.801 | 0.0 | 0.573 |
| Cranberry | 4 (219) | 0.10 (-0.27, 0.47) | 0.605 | 0.0 | 0.693 |
| Bilberry | 2 (94) | 1.40 (-0.88, 3.68) | 0.228 | 48.1 | 0.165 |
| Study design | | | | | |
| Parallel | 8 (392) | 0.12 (-0.14, 0.38) | 0.362 | 0.0 | 0.556 |
| Crossover | 2 (68) | -0.22 (-1.24, 0.81) | 0.681 | 2.4 | 0.311 |
| Duration | | | | | |
| < 8 weeks | 5 (170) | 0.27 (-0.51, 1.05) | 0.494 | 29.7 | 0.224 |
| ≥ 8 weeks | 5 (290) | 0.04 (-0.26, 0.35) | 0.786 | 0.0 | 0.878 |
| Health status of subjects | | | | | |
| Low CVD risk | 2 (63) | 0.15 (-0.14, 0.45) | 0.310 | 0.0 | 0.703 |
| High CVD risk | 8 (397) | -0.03 (-0.49, 0.43) | 0.890 | 0.0 | 0.460 |
| Type of control | | | | | |
| Placebo | 8 (342) | 0.09 (-0.18, 0.37) | 0.503 | 0.0 | 0.818 |
| Others | 2 (118) | 1.09 (-1.67, 3.84) | 0.439 | 72.4 | 0.057 |
| Study quality | | | | | |
| Low to moderate | 6 (241) | 0.12 (-0.19, 0.43) | 0.458 | 14.3 | 0.323 |
| High | 4 (219) | -0.12 (-0.97, 0.73) | 0.783 | 0.0 | 0.756 |
| Received industry funding? | | | | | |
| Yes | 6 (294) | 0.01 (-0.32, 0.35) | 0.934 | 0.0 | 0.710 |
| No | 4 (166) | 0.23 (-0.25, 0.72) | 0.346 | 21.3 | 0.282 |
| Type of intervention | | | | | |
| Powder | 6 (195) | 0.12 (-0.16, 0.40) | 0.414 | 0.0 | 0.768 |
| Beverage | 4 (265) | 0.16 (-0.90, 1.22) | 0.770 | 35.7 | 0.198 |

^1^Abbreviations: CVD, cardiovascular disease; WMD, weighted mean difference.

^2^The pooled effects sizes and 95% CIs were calculated using the random-effects model. Between-study heterogeneity was examined using the Cochrane’s Q test.

1. **Table S15** Pooled effects of purified anthocyanins and anthocyanin-rich berries on circulating C-reactive protein.^1, 2^

| Variables | Number of comparisons (subjects) | WMD (95% CI), mg/dL | *P*_difference_ | *I*^2^, % | *P*_heterogeneity_ |
| --- | --- | --- | --- | --- | --- |
| *Purified anthocyanins* | | | | | |
| Overall | 8 (579) | -0.028 (-0.050, -0.005) | 0.014 | 26.0 | 0.221 |
| Study design | | | | | |
| Parallel | 6 (497) | -0.029 (-0.053, -0.005) | 0.019 | 35.6 | 0.170 |
| Crossover | 2 (82) | 0.012 (-0.078, 0.101) | 0.800 | 0.0 | 0.644 |
| Duration | | | | | |
| < 8 weeks | 3 (200) | -0.049 (-0.061, -0.036) | <0.001 | 0.3 | 0.367 |
| ≥ 8 weeks | 5 (379) | -0.012 (-0.039, 0.016) | 0.410 | 0.0 | 0.829 |
| Health status of subjects | | | | | |
| Low CVD risk | 2 (150) | -0.049 (-0.061, -0.038) | <0.001 | 0.0 | 0.339 |
| High CVD risk | 6 (429) | -0.010 (-0.038, 0.017) | 0.461 | 0.0 | 0.863 |
| Type of control | | | | | |
| Placebo | 8 (579) | -0.028 (-0.050, -0.005) | 0.014 | 26.0 | 0.221 |
| Dose of anthocyanins | | | | | |
| ≥ 200 mg/day | 8 (579) | -0.028 (-0.050, -0.005) | 0.014 | 26.0 | 0.221 |
| Study quality | | | | | |
| Low to moderate | 1 (118) | -0.050 (-0.061, -0.039) | <0.001 | - | - |
| High | 7 (461) | -0.010 (-0.036, 0.017) | 0.476 | 0.0 | 0.925 |
| Received industry funding? | | | | | |
| Yes | 1 (50) | 0.000 (-0.053, 0.053) | 1.000 | - | - |
| No | 7 (529) | -0.037 (-0.056, -0.019) | <0.001 | 11.5 | 0.342 |
| *Anthocyanin-rich berries* | | | | | |
| Overall | 13 (655) | -0.046 (-0.070, -0.022) | <0.001 | 0.0 | 0.796 |
| Type of berry | | | | | |
| Blueberry | 5 (208) | -0.039 (-0.138, 0.061) | 0.449 | 0.0 | 0.975 |
| Cranberry | 7 (385) | -0.031 (-0.093, 0.032) | 0.337 | 14.3 | 0.321 |
| Bilberry | 1 (62) | -0.050 (-0.077, -0.023) | <0.001 | - | - |
| Study design | | | | | |
| Parallel | 11 (531) | -0.048 (-0.072, -0.023) | <0.001 | 0.0 | 0.870 |
| Crossover | 2 (124) | 0.035 (-0.176, 0.246) | 0.746 | 51.5 | 0.151 |
| Duration | | | | | |
| < 8 weeks | 4 (218) | -0.047 (-0.074, -0.021) | <0.001 | 0.0 | 0.449 |
| ≥ 8 weeks | 9 (437) | -0.040 (-0.092, 0.013) | 0.141 | 0.0 | 0.742 |
| Health status of subjects | | | | | |
| Low CVD risk | 2 (102) | -0.005 (-0.093, 0.082) | 0.906 | 0.0 | 0.679 |
| High CVD risk | 11 (553) | -0.049 (-0.074, -0.024) | <0.001 | 0.0 | 0.745 |
| Type of control | | | | | |
| Placebo | 9 (447) | -0.046 (-0.103, 0.010) | 0.107 | 0.0 | 0.592 |
| Others | 4 (208) | -0.046 (-0.072, -0.019) | 0.001 | 0.0 | 0.713 |
| Study quality | | | | | |
| Low to moderate | 7 (306) | -0.044 (-0.069, -0.020) | <0.001 | 0.0 | 0.959 |
| High | 6 (349) | -0.043 (-0.152, 0.067) | 0.444 | 18.9 | 0.290 |
| Received industry funding? | | | | | |
| Yes | 9 (465) | -0.053 (-0.120, 0.015) | 0.124 | 0.0 | 0.605 |
| No | 4 (190) | -0.045 (-0.070, -0.019) | 0.001 | 0.0 | 0.697 |
| Type of intervention | | | | | |
| Powder | 7 (280) | -0.021 (-0.077, 0.034) | 0.445 | 0.0 | 0.991 |
| Beverage | 6 (375) | -0.052 (-0.112, 0.009) | 0.096 | 18.1 | 0.296 |

^1^Abbreviations: CVD, cardiovascular disease; WMD, weighted mean difference.

^2^The pooled effects sizes and 95% CIs were calculated using the random-effects model. Between-study heterogeneity was examined using the Cochrane’s Q test.

1. **Table S16** Pooled associations of anthocyanins with incidence and mortality of coronary heart disease.^1, 2^

| Variables | Number of cohorts | RR (95% CI) | *P*_difference_ | *I*^2^, % | *P*_heterogeneity_ |
| --- | --- | --- | --- | --- | --- |
| *CHD incidence* | | | | | |
| Overall | 5 | 0.83 (0.72, 0.95) | 0.009 | 51.2 | 0.085 |
| Gender | | | | | |
| Female | 1 | 0.68 (0.49, 0.95) | 0.025 | - | - |
| Male | 1 | 0.87 (0.75, 1.01) | 0.058 | - | - |
| Both | 3 | 0.83 (0.72, 0.95) | 0.111 | 65.5 | 0.055 |
| Follow-up | | | | | |
| ≤12 years | 2 | 0.72 (0.58, 0.89) | 0.003 | 0.0 | 0.899 |
| >12 years | 3 | 0.98 (0.86, 1.12) | 0.108 | 55.2 | 0.108 |
| Location | | | | | |
| Europe | 1 | 0.71 (0.51, 0.98) | 0.040 | - | - |
| US | 4 | 0.85 (0.73, 0.98) | 0.030 | 54.1 | 0.088 |
| Study quality | | | | | |
| Low to moderate | 1 | 0.98 (0.86, 1.12) | 0.764 | - | - |
| High | 4 | 0.80 (0.71, 0.90) | <0.001 | 4.1 | 0.372 |
| *CHD mortality* | | | | | |
| Overall | 2 | 0.98 (0.79, 1.22) | 0.844 | 80.1 | 0.025 |
| Gender | | | | | |
| Female | 1 | 0.88 (0.78, 0.99) | 0.036 | - | - |
| Male | 1 | 1.10 (0.94, 1.28) | 0.226 | - | - |
| Follow-up | | | | | |
| >12 years | 2 | 0.98 (0.79, 1.22) | 0.844 | 80.1 | 0.025 |
| Location | | | | | |
| US | 2 | 0.98 (0.79, 1.22) | 0.844 | 80.1 | 0.025 |
| Study quality | | | | | |
| Low to moderate | 1 | 0.88 (0.78, 0.99) | 0.036 | - | - |
| High | 1 | 1.10 (0.94, 1.28) | 0.226 | - | - |

^1^Abbreviations: CHD, coronary heart disease; RR, relative risk.

^2^The pooled estimates and 95% CIs were calculated using the random-effects model. Between-study heterogeneity was examined using the Cochrane’s Q test.

1. **Table S17** Pooled associations of anthocyanins with incidence and mortality of total stroke.^1, 2^

| Variables | Number of cohorts | RR (95% CI) | *P*_difference_ | *I*^2^, % | *P*_heterogeneity_ |
| --- | --- | --- | --- | --- | --- |
| *Total stroke incidence* | | | | | |
| Overall | 3 | 0.84 (0.62, 1.14) | 0.256 | 91.0 | <0.001 |
| Gender | | | | | |
| Female | 1 | 0.96 (0.82, 1.12) | 0.608 | - | - |
| Male | 1 | 1.00 (0.85, 1.17) | 1.000 | - | - |
| Both | 1 | 0.61 (0.52, 0.72) | <0.001 | - | - |
| Follow-up | | | | | |
| ≤12 years | 1 | 0.61 (0.52, 0.72) | <0.001 | - | - |
| >12 years | 2 | 0.98 (0.88, 1.10) | 0.713 | 0.0 | 0.720 |
| Location | | | | | |
| Europe | 1 | 0.61 (0.52, 0.72) | <0.001 | - | - |
| US | 2 | 0.98 (0.88, 1.10) | 0.713 | 0.0 | 0.720 |
| Study quality | | | | | |
| High | 3 | 0.84 (0.62, 1.14) | 0.256 | 91.0 | <0.001 |
| *Total stroke mortality* | | | | | |
| Overall | 1 | 1.01 (0.83, 1.24) | 0.923 | - | - |
| Gender | | | | | |
| Female | 1 | 1.01 (0.83, 1.24) | 0.923 | - | - |
| Follow-up | | | | | |
| >12 years | 1 | 1.01 (0.83, 1.24) | 0.923 | - | - |
| Location | | | | | |
| US | 1 | 1.01 (0.83, 1.24) | 0.923 | - | - |
| Study quality | | | | | |
| Low to moderate | 1 | 1.01 (0.83, 1.24) | 0.923 | - | - |

^1^Abbreviations: RR, relative risk.

^2^The pooled estimates and 95% CIs were calculated using the random-effects model. Between-study heterogeneity was examined using the Cochrane’s Q test.

1. **Table S18** Pooled associations of anthocyanins with incidence of ischemic and hemorrhagic stroke.^1, 2^

| Variables | Number of cohorts | RR (95% CI) | *P*_difference_ | *I*^2^, % | *P*_heterogeneity_ |
| --- | --- | --- | --- | --- | --- |
| *Ischemic stroke incidence* | | | | | |
| Overall | 3 | 0.91 (0.78, 1.05) | 0.202 | 0.0 | 0.956 |
| Gender | | | | | |
| Female | 1 | 0.89 (0.72, 1.11) | 0.291 | - | - |
| Male | 2 | 0.93 (0.76, 1.13) | 0.447 | 0.0 | 0.869 |
| Follow-up | | | | | |
| ≤12 years | - | - | - | - | - |
| >12 years | 3 | 0.91 (0.78, 1.05) | 0.202 | 0.0 | 0.956 |
| Location | | | | | |
| Europe | 1 | 0.88 (0.47, 1.63) | 0.686 | - | - |
| US | 2 | 0.91 (0.78, 1.06) | 0.224 | 0.0 | 0.777 |
| Study quality | | | | | |
| High | 3 | 0.91 (0.78, 1.05) | 0.202 | 0.0 | 0.956 |
| *Hemorrhagic stroke incidence* | | | | | |
| Overall | 2 | 1.01 (0.75, 1.35) | 0.965 | 0.0 | 0.741 |
| Gender | | | | | |
| Female | 1 | 0.96 (0.64, 1.44) | 0.844 | - | - |
| Male | 1 | 1.06 (0.69, 1.62) | 0.787 | - | - |
| Follow-up | | | | | |
| ≤12 years | - | - | - | - | - |
| >12 years | 2 | 1.01 (0.75, 1.35) | 0.965 | 0.0 | 0.741 |
| Location | | | | | |
| US | 2 | 1.01 (0.75, 1.35) | 0.965 | 0.0 | 0.741 |
| Study quality | | | | | |
| High | 2 | 1.01 (0.75, 1.35) | 0.965 | 0.0 | 0.741 |

^1^Abbreviations: RR, relative risk.

^2^The pooled estimates and 95% CIs were calculated using the random-effects model. Between-study heterogeneity was examined using the Cochrane’s Q test.

1. **Table S19** Pooled associations of anthocyanins with incidence and mortality of total cardiovascular diseases.^1, 2^

| Variables | Number of cohorts | RR (95% CI) | *P*_difference_ | *I*^2^, % | *P*_heterogeneity_ |
| --- | --- | --- | --- | --- | --- |
| *Total CVD incidence* | | | | | |
| Overall | 4 | 0.73 (0.55, 0.97) | 0.030 | 76.7 | 0.005 |
| Gender | | | | | |
| Both | 4 | 0.73 (0.55, 0.97) | 0.030 | 76.7 | 0.005 |
| Follow-up | | | | | |
| ≤12 years | 3 | 0.64 (0.53, 0.78) | <0.001 | 0.0 | 0.807 |
| >12 years | 1 | 0.95 (0.86, 1.05) | 0.314 | - | - |
| Location | | | | | |
| Europe | 3 | 0.64 (0.53, 0.78) | <0.001 | 0.0 | 0.807 |
| US | 1 | 0.95 (0.86, 1.05) | 0.314 | - | - |
| Study quality | | | | | |
| Low to moderate | 1 | 0.95 (0.86, 1.05) | 0.314 | - | - |
| High | 3 | 0.64 (0.53, 0.78) | <0.001 | 0.0 | 0.807 |
| *Total CVD mortality* | | | | | |
| Overall | 9 | 0.91 (0.87, 0.96) | <0.001 | 0.0 | 0.712 |
| Gender | | | | | |
| Female | 3 | 0.89 (0.82, 0.96) | 0.003 | 0.0 | 0.435 |
| Male | 2 | 0.92 (0.79, 1.07) | 0.263 | 0.0 | 0.772 |
| Both | 4 | 0.93 (0.88, 0.99) | 0.014 | 0.0 | 0.405 |
| Follow-up | | | | | |
| ≤12 years | 6 | 0.85 (0.76, 0.96) | 0.007 | 0.0 | 0.579 |
| >12 years | 3 | 0.93 (0.88, 0.97) | 0.002 | 0.0 | 0.758 |
| Location | | | | | |
| Europe | 3 | 0.95 (0.88, 1.03) | 0.234 | 0.0 | 0.464 |
| US | 3 | 0.90 (0.83, 0.96) | 0.002 | 0.0 | 0.582 |
| Australia | 3 | 0.89 (0.81, 0.98) | 0.012 | 0.0 | 0.620 |
| Study quality | | | | | |
| Low to moderate | 2 | 0.91 (0.83, 0.99) | 0.026 | 0.0 | 0.395 |
| High | 7 | 0.92 (0.87, 0.97) | 0.001 | 0.0 | 0.591 |

^1^Abbreviations: CVD, cardiovascular disease; RR, relative risk.

^2^The pooled estimates and 95% CIs were calculated using the random-effects model. Between-study heterogeneity was examined using the Cochrane’s Q test.

**Supplemental references**

[Dataset] (2018). *United States Department of Agriculture Database for the Flavonoid Content of Selected Foods, Release 3.3* Available: <https://www.ars.usda.gov/ARSUserFiles/80400535/Data/Flav/Flav3.3.pdf>.

Adriouch, S., Lampuré, A., Nechba, A., Baudry, J., Assmann, K., Kesse-Guyot, E., et al. (2018). Prospective Association between Total and Specific Dietary Polyphenol Intakes and Cardiovascular Disease Risk in the Nutrinet-Santé French Cohort. *Nutrients* 10(11). doi: 10.3390/nu10111587.

Alnajjar, M., Barik, S., Bestwick, C., Campbell, F., Cruickshank, M., Farquharson, F., et al. (2019). Anthocyanin-enriched bilberry extract attenuates glycaemic response in overweight volunteers without changes in insulin. *Journal of Functional Foods* 64**,** 103597. doi: 10.1016/j.jff.2019.103597.

Arevström, L., Bergh, C., Landberg, R., Wu, H., Rodriguez-Mateos, A., Waldenborg, M., et al. (2019). Freeze-dried bilberry (Vaccinium myrtillus) dietary supplement improves walking distance and lipids after myocardial infarction: an open-label randomized clinical trial. *Nutr Res* 62**,** 13-22. doi: 10.1016/j.nutres.2018.11.008.

Basu, A., Betts, N.M., Ortiz, J., Simmons, B., Wu, M., and Lyons, T.J. (2011). Low-energy cranberry juice decreases lipid oxidation and increases plasma antioxidant capacity in women with metabolic syndrome. *Nutr Res* 31(3)**,** 190-196. doi: 10.1016/j.nutres.2011.02.003.

Basu, A., Du, M., Leyva, M.J., Sanchez, K., Betts, N.M., Wu, M., et al. (2010). Blueberries decrease cardiovascular risk factors in obese men and women with metabolic syndrome. *J Nutr* 140(9)**,** 1582-1587. doi: 10.3945/jn.110.124701.

Bondonno, N.P., Dalgaard, F., Kyro, C., Murray, K., Bondonno, C.P., Lewis, J.R., et al. (2019). Flavonoid intake is associated with lower mortality in the Danish Diet Cancer and Health Cohort. *Nat Commun* 10(1)**,** 3651. doi: 10.1038/s41467-019-11622-x.

Bondonno, N.P., Lewis, J.R., Blekkenhorst, L.C., Bondonno, C.P., Shin, J.H., Croft, K.D., et al. (2020). Association of flavonoids and flavonoid-rich foods with all-cause mortality: The Blue Mountains Eye Study. *Clinical nutrition (Edinburgh, Scotland)* 39(1)**,** 141-150. doi: 10.1016/j.clnu.2019.01.004.

Cassidy, A., Bertoia, M., Chiuve, S., Flint, A., Forman, J., and Rimm, E.B. (2016). Habitual intake of anthocyanins and flavanones and risk of cardiovascular disease in men. *The American journal of clinical nutrition* 104(3)**,** 587-594. doi: 10.3945/ajcn.116.133132.

Cassidy, A., Mukamal, K.J., Liu, L., Franz, M., Eliassen, A.H., and Rimm, E.B. (2013). High anthocyanin intake is associated with a reduced risk of myocardial infarction in young and middle-aged women. *Circulation* 127(2)**,** 188-196. doi: 10.1161/circulationaha.112.122408.

Cassidy, A., Rimm, E.B., O'Reilly, E.J., Logroscino, G., Kay, C., Chiuve, S.E., et al. (2012). Dietary flavonoids and risk of stroke in women. *Stroke* 43(4)**,** 946-951. doi: 10.1161/strokeaha.111.637835.

Chew, B., Mathison, B., Kimble, L., McKay, D., Kaspar, K., Khoo, C., et al. (2019). Chronic consumption of a low calorie, high polyphenol cranberry beverage attenuates inflammation and improves glucoregulation and HDL cholesterol in healthy overweight humans: a randomized controlled trial. *Eur J Nutr* 58(3)**,** 1223-1235. doi: 10.1007/s00394-018-1643-z.

Curtis, P.J., Kroon, P.A., Hollands, W.J., Walls, R., Jenkins, G., Kay, C.D., et al. (2009). Cardiovascular disease risk biomarkers and liver and kidney function are not altered in postmenopausal women after ingesting an elderberry extract rich in anthocyanins for 12 weeks. *J Nutr* 139(12)**,** 2266-2271. doi: 10.3945/jn.109.113126.

Curtis, P.J., van der Velpen, V., Berends, L., Jennings, A., Feelisch, M., Umpleby, A.M., et al. (2019). Blueberries improve biomarkers of cardiometabolic function in participants with metabolic syndrome-results from a 6-month, double-blind, randomized controlled trial. *Am J Clin Nutr* 109(6)**,** 1535-1545. doi: 10.1093/ajcn/nqy380.

Dohadwala, M.M., Holbrook, M., Hamburg, N.M., Shenouda, S.M., Chung, W.B., Titas, M., et al. (2011). Effects of cranberry juice consumption on vascular function in patients with coronary artery disease. *Am J Clin Nutr* 93(5)**,** 934-940. doi: 10.3945/ajcn.110.004242.

Du, C., Smith, A., Avalos, M., South, S., Crabtree, K., Wang, W., et al. (2019). Blueberries Improve Pain, Gait Performance, and Inflammation in Individuals with Symptomatic Knee Osteoarthritis. *Nutrients* 11(2). doi: 10.3390/nu11020290.

Duthie, S.J., Jenkinson, A.M., Crozier, A., Mullen, W., Pirie, L., Kyle, J., et al. (2006). The effects of cranberry juice consumption on antioxidant status and biomarkers relating to heart disease and cancer in healthy human volunteers. *Eur J Nutr* 45(2)**,** 113-122. doi: 10.1007/s00394-005-0572-9.

Flammer, A.J., Martin, E.A., Gössl, M., Widmer, R.J., Lennon, R.J., Sexton, J.A., et al. (2013). Polyphenol-rich cranberry juice has a neutral effect on endothelial function but decreases the fraction of osteocalcin-expressing endothelial progenitor cells. *Eur J Nutr* 52(1)**,** 289-296. doi: 10.1007/s00394-012-0334-4.

Goetz, M.E., Judd, S.E., Safford, M.M., Hartman, T.J., McClellan, W.M., and Vaccarino, V. (2016). Dietary flavonoid intake and incident coronary heart disease: the REasons for Geographic and Racial Differences in Stroke (REGARDS) study. *Am J Clin Nutr* 104(5)**,** 1236-1244. doi: 10.3945/ajcn.115.129452.

Guo, Y., Zhang, P., Liu, Y., Zha, L., Ling, W., and Guo, H. (2020). A dose-response evaluation of purified anthocyanins on inflammatory and oxidative biomarkers and metabolic risk factors in healthy young adults: A randomized controlled trial. *Nutrition* 74**,** 110745. doi: 10.1016/j.nut.2020.110745.

Hassellund, S.S., Flaa, A., Sandvik, L., Kjeldsen, S.E., and Rostrup, M. (2012). Effects of anthocyanins on blood pressure and stress reactivity: a double-blind randomized placebo-controlled crossover study. *J Hum Hypertens* 26(6)**,** 396-404. doi: 10.1038/jhh.2011.41.

Hsia, D.S., Zhang, D.J., Beyl, R.S., Greenway, F.L., and Khoo, C. (2020). Effect of daily consumption of cranberry beverage on insulin sensitivity and modification of cardiovascular risk factors in adults with obesity: a pilot, randomised, placebo-controlled study. *Br J Nutr***,** 1-9. doi: 10.1017/s0007114520001336.

Ivey, K.L., Lewis, J.R., Prince, R.L., and Hodgson, J.M. (2013). Tea and non-tea flavonol intakes in relation to atherosclerotic vascular disease mortality in older women. *Br J Nutr* 110(9)**,** 1648-1655. doi: 10.1017/s0007114513000780.

Jacques, P.F., Cassidy, A., Rogers, G., Peterson, J.J., and Dwyer, J.T. (2015). Dietary flavonoid intakes and CVD incidence in the Framingham Offspring Cohort. *Br J Nutr* 114(9)**,** 1496-1503. doi: 10.1017/s0007114515003141.

Johnson, S.A., Feresin, R.G., Navaei, N., Figueroa, A., Elam, M.L., Akhavan, N.S., et al. (2017). Effects of daily blueberry consumption on circulating biomarkers of oxidative stress, inflammation, and antioxidant defense in postmenopausal women with pre- and stage 1-hypertension: a randomized controlled trial. *Food Funct* 8(1)**,** 372-380. doi: 10.1039/c6fo01216g.

Johnson, S.A., Figueroa, A., Navaei, N., Wong, A., Kalfon, R., Ormsbee, L.T., et al. (2015). Daily blueberry consumption improves blood pressure and arterial stiffness in postmenopausal women with pre- and stage 1-hypertension: a randomized, double-blind, placebo-controlled clinical trial. *J Acad Nutr Diet* 115(3)**,** 369-377. doi: 10.1016/j.jand.2014.11.001.

Karlsen, A., Paur, I., Bøhn, S.K., Sakhi, A.K., Borge, G.I., Serafini, M., et al. (2010). Bilberry juice modulates plasma concentration of NF-kappaB related inflammatory markers in subjects at increased risk of CVD. *Eur J Nutr* 49(6)**,** 345-355. doi: 10.1007/s00394-010-0092-0.

Karlsen, A., Retterstøl, L., Laake, P., Paur, I., Bøhn, S.K., Sandvik, L., et al. (2007). Anthocyanins inhibit nuclear factor-kappaB activation in monocytes and reduce plasma concentrations of pro-inflammatory mediators in healthy adults. *J Nutr* 137(8)**,** 1951-1954. doi: 10.1093/jn/137.8.1951.

Khan, F., Ray, S., Craigie, A.M., Kennedy, G., Hill, A., Barton, K.L., et al. (2014). Lowering of oxidative stress improves endothelial function in healthy subjects with habitually low intake of fruit and vegetables: a randomized controlled trial of antioxidant- and polyphenol-rich blackcurrant juice. *Free Radic Biol Med* 72**,** 232-237. doi: 10.1016/j.freeradbiomed.2014.04.006.

Lee, I.T., Chan, Y.C., Lin, C.W., Lee, W.J., and Sheu, W.H. (2008). Effect of cranberry extracts on lipid profiles in subjects with Type 2 diabetes. *Diabet Med* 25(12)**,** 1473-1477. doi: 10.1111/j.1464-5491.2008.02588.x.

Li, D., Zhang, Y., Liu, Y., Sun, R., and Xia, M. (2015). Purified anthocyanin supplementation reduces dyslipidemia, enhances antioxidant capacity, and prevents insulin resistance in diabetic patients. *J Nutr* 145(4)**,** 742-748. doi: 10.3945/jn.114.205674.

McAnulty, L.S., Collier, S.R., Landram, M.J., Whittaker, D.S., Isaacs, S.E., Klemka, J.M., et al. (2014). Six weeks daily ingestion of whole blueberry powder increases natural killer cell counts and reduces arterial stiffness in sedentary males and females. *Nutr Res* 34(7)**,** 577-584. doi: 10.1016/j.nutres.2014.07.002.

McAnulty, S.R., McAnulty, L.S., Morrow, J.D., Khardouni, D., Shooter, L., Monk, J., et al. (2005). Effect of daily fruit ingestion on angiotensin converting enzyme activity, blood pressure, and oxidative stress in chronic smokers. *Free Radic Res* 39(11)**,** 1241-1248. doi: 10.1080/10715760500306836.

McCullough, M.L., Peterson, J.J., Patel, R., Jacques, P.F., Shah, R., and Dwyer, J.T. (2012). Flavonoid intake and cardiovascular disease mortality in a prospective cohort of US adults. *Am J Clin Nutr* 95(2)**,** 454-464. doi: 10.3945/ajcn.111.016634.

Mink, P.J., Scrafford, C.G., Barraj, L.M., Harnack, L., Hong, C.P., Nettleton, J.A., et al. (2007). Flavonoid intake and cardiovascular disease mortality: a prospective study in postmenopausal women. *Am J Clin Nutr* 85(3)**,** 895-909. doi: 10.1093/ajcn/85.3.895.

Mursu, J., Voutilainen, S., Nurmi, T., Tuomainen, T.-P., Kurl, S., and Salonen, J.T. (2008). Flavonoid intake and the risk of ischaemic stroke and CVD mortality in middle-aged Finnish men: the Kuopio Ischaemic Heart Disease Risk Factor Study. *The British journal of nutrition* 100(4)**,** 890-895. doi: 10.1017/S0007114508945694.

Novotny, J.A., Baer, D.J., Khoo, C., Gebauer, S.K., and Charron, C.S. (2015). Cranberry juice consumption lowers markers of cardiometabolic risk, including blood pressure and circulating C-reactive protein, triglyceride, and glucose concentrations in adults. *J Nutr* 145(6)**,** 1185-1193. doi: 10.3945/jn.114.203190.

Nyberg, S., Gerring, E., Gjellan, S., Vergara, M., Lindström, T., and Nystrom, F.H. (2013). Effects of exercise with or without blueberries in the diet on cardio-metabolic risk factors: an exploratory pilot study in healthy subjects. *Ups J Med Sci* 118(4)**,** 247-255. doi: 10.3109/03009734.2013.825348.

Ohguro, H., Ohguro, I., Katai, M., and Tanaka, S. (2012). Two-year randomized, placebo-controlled study of black currant anthocyanins on visual field in glaucoma. *Ophthalmologica* 228(1)**,** 26-35. doi: 10.1159/000335961.

Ponzo, V., Goitre, I., Fadda, M., Gambino, R., De Francesco, A., Soldati, L., et al. (2015). Dietary flavonoid intake and cardiovascular risk: a population-based cohort study. *J Transl Med* 13**,** 218. doi: 10.1186/s12967-015-0573-2.

Qin, Y., Xia, M., Ma, J., Hao, Y., Liu, J., Mou, H., et al. (2009). Anthocyanin supplementation improves serum LDL- and HDL-cholesterol concentrations associated with the inhibition of cholesteryl ester transfer protein in dyslipidemic subjects. *Am J Clin Nutr* 90(3)**,** 485-492. doi: 10.3945/ajcn.2009.27814.

Riso, P., Klimis-Zacas, D., Del Bo, C., Martini, D., Campolo, J., Vendrame, S., et al. (2013). Effect of a wild blueberry (Vaccinium angustifolium) drink intervention on markers of oxidative stress, inflammation and endothelial function in humans with cardiovascular risk factors. *Eur J Nutr* 52(3)**,** 949-961. doi: 10.1007/s00394-012-0402-9.

Rodriguez-Mateos, A., Istas, G., Boschek, L., Feliciano, R.P., Mills, C.E., Boby, C., et al. (2019). Circulating Anthocyanin Metabolites Mediate Vascular Benefits of Blueberries: Insights From Randomized Controlled Trials, Metabolomics, and Nutrigenomics. *J Gerontol A Biol Sci Med Sci* 74(7)**,** 967-976. doi: 10.1093/gerona/glz047.

Simão, T.N., Lozovoy, M.A., Simão, A.N., Oliveira, S.R., Venturini, D., Morimoto, H.K., et al. (2013). Reduced-energy cranberry juice increases folic acid and adiponectin and reduces homocysteine and oxidative stress in patients with the metabolic syndrome. *Br J Nutr* 110(10)**,** 1885-1894. doi: 10.1017/s0007114513001207.

Skarpańska-Stejnborn, A., Basta, P., Trzeciak, J., Michalska, A., Kafkas, M.E., and Woitas-Ślubowska, D. (2017). Effects of cranberry (Vaccinum macrocarpon) supplementation on iron status and inflammatory markers in rowers. *J Int Soc Sports Nutr* 14**,** 7. doi: 10.1186/s12970-017-0165-z.

Stote, K.S., Wilson, M.M., Hallenbeck, D., Thomas, K., Rourke, J.M., Sweeney, M.I., et al. (2020). Effect of Blueberry Consumption on Cardiometabolic Health Parameters in Men with Type 2 Diabetes: An 8-Week, Double-Blind, Randomized, Placebo-Controlled Trial. *Curr Dev Nutr* 4(4)**,** nzaa030. doi: 10.1093/cdn/nzaa030.

Stull, A.J., Cash, K.C., Champagne, C.M., Gupta, A.K., Boston, R., Beyl, R.A., et al. (2015). Blueberries improve endothelial function, but not blood pressure, in adults with metabolic syndrome: a randomized, double-blind, placebo-controlled clinical trial. *Nutrients* 7(6)**,** 4107-4123. doi: 10.3390/nu7064107.

Stull, A.J., Cash, K.C., Johnson, W.D., Champagne, C.M., and Cefalu, W.T. (2010). Bioactives in blueberries improve insulin sensitivity in obese, insulin-resistant men and women. *J Nutr* 140(10)**,** 1764-1768. doi: 10.3945/jn.110.125336.

Thompson, K., Hosking, H., Pederick, W., Singh, I., and Santhakumar, A.B. (2017a). The effect of anthocyanin supplementation in modulating platelet function in sedentary population: a randomised, double-blind, placebo-controlled, cross-over trial. *Br J Nutr* 118(5)**,** 368-374. doi: 10.1017/s0007114517002124.

Thompson, K., Pederick, W., Singh, I., and Santhakumar, A.B. (2017b). Anthocyanin supplementation in alleviating thrombogenesis in overweight and obese population: A randomized, double-blind, placebo-controlled study. *Journal of Functional Foods* 32**,** 131-138. doi: 10.1016/j.jff.2017.02.031.

Tresserra-Rimbau, A., Rimm, E.B., Medina-Remón, A., Martínez-González, M.A., de la Torre, R., Corella, D., et al. (2014). Inverse association between habitual polyphenol intake and incidence of cardiovascular events in the PREDIMED study. *Nutr Metab Cardiovasc Dis* 24(6)**,** 639-647. doi: 10.1016/j.numecd.2013.12.014.

Vidlar, A., Vostalova, J., Ulrichova, J., Student, V., Stejskal, D., Reichenbach, R., et al. (2010). The effectiveness of dried cranberries ( Vaccinium macrocarpon) in men with lower urinary tract symptoms. *Br J Nutr* 104(8)**,** 1181-1189. doi: 10.1017/s0007114510002059.

Xu, Z., Xie, J., Zhang, H., Pang, J., Li, Q., Wang, X., et al. (2020). Anthocyanin supplementation at different doses improves cholesterol efflux capacity in subjects with dyslipidemia-a randomized controlled trial. *Eur J Clin Nutr*. doi: 10.1038/s41430-020-0609-4.

Yang, L., Ling, W., Yang, Y., Chen, Y., Tian, Z., Du, Z., et al. (2017). Role of Purified Anthocyanins in Improving Cardiometabolic Risk Factors in Chinese Men and Women with Prediabetes or Early Untreated Diabetes-A Randomized Controlled Trial. *Nutrients* 9(10). doi: 10.3390/nu9101104.

Zamora-Ros, R., Jiménez, C., Cleries, R., Agudo, A., Sánchez, M.J., Sánchez-Cantalejo, E., et al. (2013). Dietary flavonoid and lignan intake and mortality in a Spanish cohort. *Epidemiology* 24(5)**,** 726-733. doi: 10.1097/EDE.0b013e31829d5902.

Zare Javid, A., Maghsoumi-Norouzabad, L., Ashrafzadeh, E., Yousefimanesh, H.A., Zakerkish, M., Ahmadi Angali, K., et al. (2018). Impact of Cranberry Juice Enriched with Omega-3 Fatty Acids Adjunct with Nonsurgical Periodontal Treatment on Metabolic Control and Periodontal Status in Type 2 Patients with Diabetes with Periodontal Disease. *J Am Coll Nutr* 37(1)**,** 71-79. doi: 10.1080/07315724.2017.1357509.

Zhang, H., Xu, Z., Zhao, H., Wang, X., Pang, J., Li, Q., et al. (2020). Anthocyanin supplementation improves anti-oxidative and anti-inflammatory capacity in a dose–response manner in subjects with dyslipidemia. *Redox Biol* 32**,** 101474. doi: 10.1016/j.redox.2020.101474.

Zhang, P.W., Chen, F.X., Li, D., Ling, W.H., and Guo, H.H. (2015). A CONSORT-compliant, randomized, double-blind, placebo-controlled pilot trial of purified anthocyanin in patients with nonalcoholic fatty liver disease. *Medicine (Baltimore)* 94(20)**,** e758. doi: 10.1097/md.0000000000000758.

Zhu, Y., Ling, W., Guo, H., Song, F., Ye, Q., Zou, T., et al. (2013). Anti-inflammatory effect of purified dietary anthocyanin in adults with hypercholesterolemia: a randomized controlled trial. *Nutr Metab Cardiovasc Dis* 23(9)**,** 843-849. doi: 10.1016/j.numecd.2012.06.005.

Zhu, Y., Xia, M., Yang, Y., Liu, F., Li, Z., Hao, Y., et al. (2011). Purified anthocyanin supplementation improves endothelial function via NO-cGMP activation in hypercholesterolemic individuals. *Clin Chem* 57(11)**,** 1524-1533. doi: 10.1373/clinchem.2011.167361.
